# A New Role for Translation Initiation Factor 2 in Maintaining Genome Integrity

**DOI:** 10.1371/journal.pgen.1002648

**Published:** 2012-04-19

**Authors:** K. Elizabeth Madison, Mona R. Abdelmeguid, Erica N. Jones-Foster, Hiroshi Nakai

**Affiliations:** Department of Biochemistry and Molecular and Cellular Biology, Georgetown University Medical Center, Washington, D.C, United States of America; Uppsala University, Sweden

## Abstract

*Escherichia coli* translation initiation factor 2 (IF2) performs the unexpected function of promoting transition from recombination to replication during bacteriophage Mu transposition *in vitro*, leading to initiation by replication restart proteins. This function has suggested a role of IF2 in engaging cellular restart mechanisms and regulating the maintenance of genome integrity. To examine the potential effect of IF2 on restart mechanisms, we characterized its influence on cellular recovery following DNA damage by methyl methanesulfonate (MMS) and UV damage. Mutations that prevent expression of full-length IF2-1 or truncated IF2-2 and IF2-3 isoforms affected cellular growth or recovery following DNA damage differently, influencing different restart mechanisms. A deletion mutant (*del1*) expressing only IF2-2/3 was severely sensitive to growth in the presence of DNA-damaging agent MMS. Proficient as wild type in repairing DNA lesions and promoting replication restart upon removal of MMS, this mutant was nevertheless unable to sustain cell growth in the presence of MMS; however, growth in MMS could be partly restored by disruption of *sulA*, which encodes a cell division inhibitor induced during replication fork arrest. Moreover, such characteristics of *del1* MMS sensitivity were shared by restart mutant *priA300*, which encodes a helicase-deficient restart protein. Epistasis analysis indicated that *del1* in combination with *priA300* had no further effects on cellular recovery from MMS and UV treatment; however, the *del2/3* mutation, which allows expression of only IF2-1, synergistically increased UV sensitivity in combination with *priA300*. The results indicate that full-length IF2, in a function distinct from truncated forms, influences the engagement or activity of restart functions dependent on PriA helicase, allowing cellular growth when a DNA–damaging agent is present.

## Author Summary

Translation Initiation Factor 2 (IF2) is a bacterial protein that plays an essential role in the initiation of protein synthesis. As such, it not only has an important influence on cellular growth but also is subject to regulation in response to physiological conditions such as nutritional deprivation. Biochemical characterization of IF2's function in replicating movable genetic elements has suggested a new role in the maintenance of genome integrity, potentially regulating replication restart. The parasitic elements exploit the cellular replication restart system to duplicate themselves as they transpose to new positions of the chromosome. In this process, IF2 makes way for action of restart proteins, which assemble replication enzymes for initiation of DNA synthesis. For the bacterial cell, the restart system is the means by which it copes with accidents that result in arrest of chromosomal replication, promoting resumption of replication. We present evidence for an IF2 function associated with restart proteins, allowing chromosomal replication in the presence of DNA–damaging agents. As the IF2 function is a highly conserved one found in all organisms, the findings have implications for understanding the maintenance of genome integrity with respect to physiological status, which can be sensed by the translation apparatus.

## Introduction

Translation Initiation Factor 2 (IF2; for a review, see [1]) is an essential cellular protein that brings mRNA, the 30S ribosome, and the initiator fMet-tRNA together into the 30S initiation complex and then promotes association with the 50S ribosomal unit to form the 70S initiation complex [2]–[4]. We have previously identified it as an essential component for reconstituting bacteriophage Mu replication by transposition *in vitro*, a process in which IF2 makes way for initiation of DNA synthesis by the cellular restart proteins [5]. This finding raises the question whether IF2 could play an important function in the maintenance of genome integrity by regulating the engagement or activity of restart proteins.

**Figure 1:**
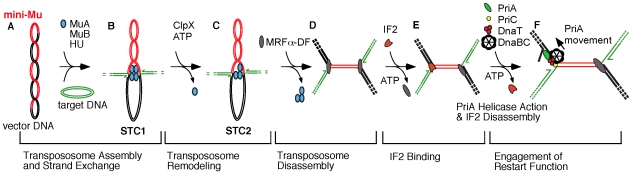
Transition from transpososome to replisome during bacteriophage Mu transposition. The model reflects changes in nucleoprotein complexes at the Mu ends as the transpososome, assembled from MuA protomers, is sequentially remodeled to a replisome [5], [9], [15]. A) A supercoiled plasmid bearing a miniature version of the Mu genome serves as the donor for transposition *in vitro*; a second plasmid is used as the target for transposition. B) The phage-encoded MuA is assembled into an oligomeric transpososome, tightly bound to the Mu ends, and this transpososome-DNA complex is preserved as it catalyzes the transfer of Mu ends to target DNA, forming a DNA fork at each Mu end (Strand Transfer Complex 1 or STC1). The half arrows depict the 3′-OH ends of DNA at each fork, which is a potential site for initiation of Mu DNA replication. C) ClpX remodels the transpososome (STC2), weakening its interaction with DNA [14], [15] and preparing the complex for disassembly. D) An unknown host factor (MRFα-DF) completes transpososome disassembly, forming a new nucleoprotein assembly that still does not permit access of the Mu fork to replication and restart proteins. E) IF2 binds to Mu DNA and unlocks the replication block at one or both forks. F) PriA binds to the Mu fork, and its helicase action promotes the disassembly of IF2, leading to the loading of the major replicative helicase DnaB from the DnaB-DnaC complex for replisome assembly. The indicated movement of PriA on the lagging strand template may serve the dual function of promoting IF2 disassembly and unwinding the DNA helix for DnaB loading [5], [29].

For bacteriophage Mu transposition *in vitro* [6], IF2 plays a critical part [5] during the transition from strand exchange catalyzed by MuA transposase [7], [8] to the assembly of the replisome by the host replication restart proteins [9] (Figure 1; for a review, see [10]). IF2 binds to Mu DNA only upon disassembly of the oligomeric MuA transpososome that remains tightly bound to Mu ends after strand exchange [11]–13. This process begins as ClpX weakens the transpososome assembly [14]–[16] and is completed by host factors which promote transition to replisome assembly [5], [9], [15], [17]. Strand exchange creates a fork at each Mu end, creating a potential site for initiating Mu DNA replication. However, the Mu forks retain a block to initiation of DNA replication even after transpososome disassembly, and IF2 appears to play a key role in unlocking this complex [5]. Restart proteins are subsequently assembled, beginning with the displacement of the IF2 by PriA helicase. The reaction *in vitro* specifically requires the *E. coli* replication restart proteins PriA, PriC, and DnaT but not PriB, indicating that the mode of Mu replication reconstituted in this system is through the PriA-PriC restart system [18], [19]. (The PriA-PriC pathway is one of the two major cellular restart pathways, the other being the PriA-PriB pathway, which requires PriA, PriB, and DnaT [18].) Additionally, only truncated forms of IF2 (IF2-2 and IF2-3; M_r_ of 79.7 and 78.8 k compared to 97.3 k for full-length IF2-1), synthesized from two internal, in-frame start codons within the *infB* gene, have been found to be active in this *in vitro* system.

Indeed, the role of the various IF2 forms in translation remains unclear. Full-length (IF2-1) and truncated (IF2-2/3) forms are present in nearly equimolar amounts under normal growth conditions [20], [21], and IF2-2/3 levels increase with respect to IF2-1 during cold shock [22]. Mutations that prevent expression of IF2-1 or IF2-2/3 elicit cold sensitivity [21]. However, even IF2 with one-third of its residues deleted from the N-terminal end has intact activities *in vitro* as translation factor and supports cell viability when present in excess [23], [24].

IF2's role in Mu DNA replication by transposition *in vitro* raises the question whether it can influence or regulate the engagement of cellular restart mechanisms. The apparent function implied by the Mu replication system is that by binding to forked DNA templates, it may promote or regulate the action of restart proteins. IF2's molecular chaperone activity [25] potentially plays a function similar to ClpX, promoting remodeling of the nucleoprotein assembly at the Mu ends for the transition to a new complex [5] or plays a key part in the activation of enzymatic functions necessary for replication restart. Moreover, IF2's major function as translation factor as well as its possible function as a transcriptional activator [26], [27] also indicate its potential to influence restart mechanisms by promoting expression of proteins needed for this process. Indeed, the role of IF2 in Mu replication may be an idiosyncrasy of Mu as a parasite exploiting host proteins to promote its own propagation; alternatively, it may reflect IF2's cellular role in regulating engagement of restart functions, a function that Mu exploits as a parasite.

In this work, we examined whether IF2 function can affect specific pathways for replication restart by perturbing its function with mutations that prevent expression of IF2-1 or IF2-2/3. Only truncated forms of IF2 have been found to be active in the reconstituted Mu replication system by the PriA-PriC pathway [5]. While this result does not necessarily indicate that only the truncated forms of IF2 may be involved in restart mechanisms (the *in vitro* system may have lacked factors needed to engage IF2-1), it nevertheless suggests functional differences between isoforms that may be examined *in vivo*.

Here, we demonstrate that the loss of IF2-1 or IF2-2/3 results in different defects in restart mechanisms that cope with DNA damage during cell growth. In particular, the loss of IF2-1 elicits a phenotype that is analogous to a certain restart mutant. No matter the mechanism by which IF2 influences restart mechanisms, the results indicate a new function of IF2 in influencing the engagement of restart mechanisms, the relative levels of IF2 isoforms having the potential to affect the choice or course of the restart mechanism. We discuss the potential for IF2 to regulate maintenance of genome integrity with respect to cell physiology, suggesting a means for coordinating replication, recombination, and repair with translation status.

## Results

### IF2 binds to Mu ends *in vivo* upon induction of Mu development

In the *in vitro* Mu replication system, binding of IF2-2 can be detected after strand exchange just prior to the binding of the restart protein PriA [5]. Since this is the major basis for suspecting that IF2 may serve a function that affects activity of restart functions, we wished to confirm that IF2 indeed binds at or near Mu ends *in vivo* when Mu development is induced. Chromatin immunoprecipitation (ChIP) analysis was conducted with extracts of induced lysogens expressing IF2 with an N-terminal S tag (S-IF2) after extensive RNase treatment.

**Figure 2:**
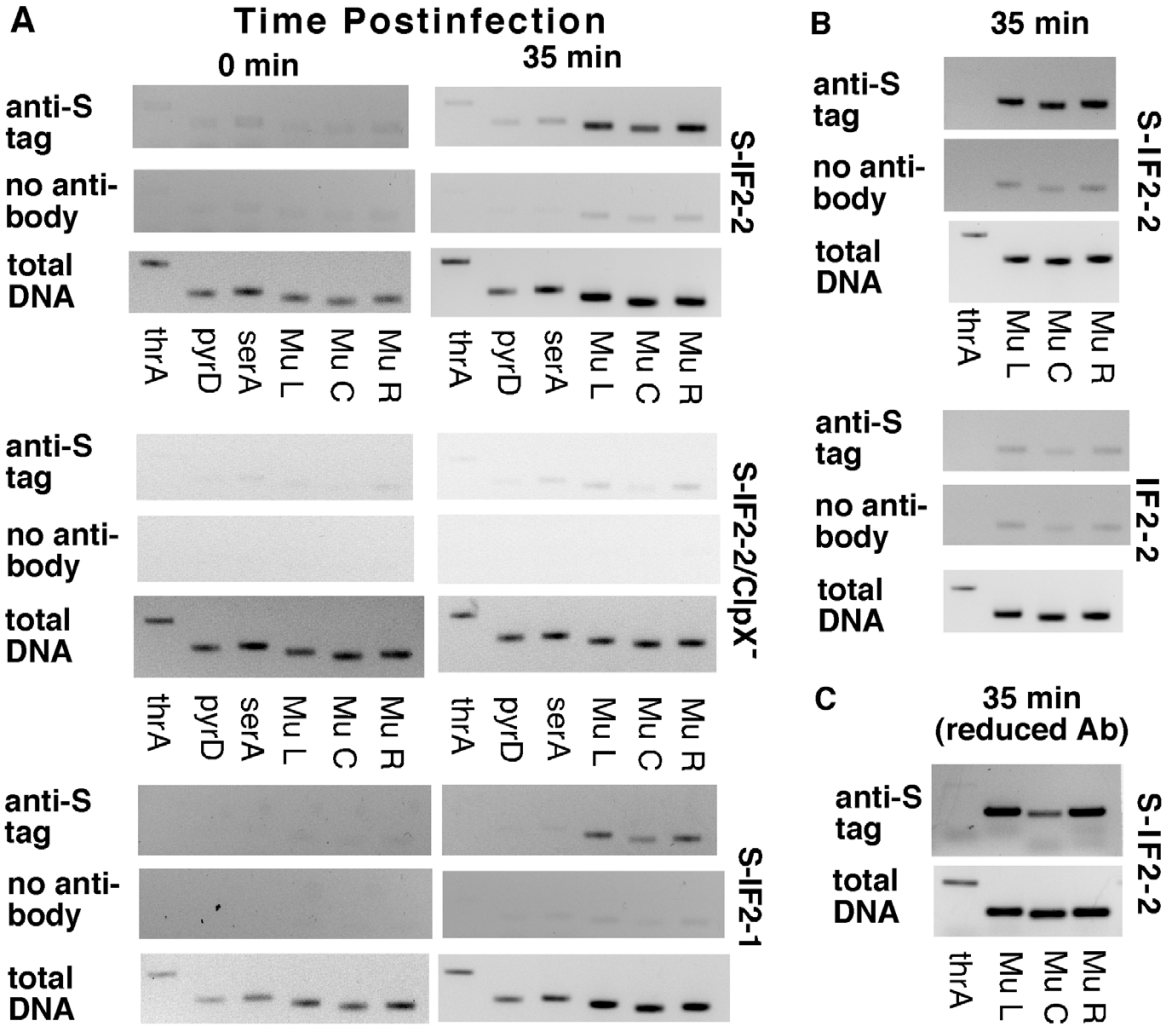
Binding of S-IF2 to Mu ends upon induction of phage replication by transposition. Mu development in GTN373 (*his::*Mu*cts62 priA300 del(gpt-lac)5*) and GTN622 (*his::*Mu*cts62 priA300 clpX::kan del(gpt-lac)5*) transformed with pBAD24-S-IF2-1, pBAD24-S-IF2-2, or pBAD24-IF2-2 were induced by incubation at 42°C, and immunoprecipitation with anti-S-tag monoclonal antibody was conducted with samples from 0 and 35 min postinduction. The presence of host and Mu DNA sequences in immunocomplexes were detected by amplifying 200–400 bp segments of template DNA (2 µl of indicated DNA dilutions in 10-µl reactions) in a 26-cycle PCR, using primers for amplifying *thrA*, *pyrD*, *serA*, Mu left end (L), Mu center (C), and Mu right end (R). A) Pull-down of Mu sequences by anti-S-tag antibody during Mu development. 1:20 dilutions of the immunoprecipitation (IP) and the no antibody control and 1∶2500 dilutions of total DNA were used. B) Comparison of IP with S-tagged and untagged IF2-2. GTN373 transformed with the indicated plasmids were used. C) Binding of S-IF2-2 concentrated at or near Mu ends. Analysis was conducted with induced pBAD24-S-IF2-2/GTN373, using one-fifth the standard amount of antibody.

Mu DNA was co-precipitated with S-IF2-1 and S-IF2-2 in induced GTN373 (a thermoinducible Mu lysogen) at 35 min postinduction (Figure 2A, S-IF2-1 and S-IF2-2), using antibody against the S tag. In contrast, relatively little Mu DNA was precipitated with S-IF2-2 upon inducing the isogenic lysogen that has a *clpX* knockout mutation (Figure 2A, S-IF2-2 ClpX^−^) and thus cannot support Mu replication [28]. This result parallels findings *in vitro* that the omission of molecular chaperone ClpX from the reaction system does not permit binding of IF2-2 to Mu DNA and the initiation of Mu replication [5], [15]. As it appeared that Mu ends were being enriched in immunoprecipitations when cells were undergoing Mu replication, we repeated the ChIP with 5-fold less antibody to ascertain whether bound S-IF2-2 in induced GTN373 is concentrated around Mu ends. In the immunoprecipitated samples, the Mu ends sequences were enriched over the center sequences (18 kb from either end) as well as host DNA (Figure 2C). In the control PCR amplification of total DNA, the Mu end and center sequences were amplified to the same extent. Mu PCR products were produced at higher levels than the host *thrA* PCR product at 35 min postinduction, reflecting the replication of Mu during lytic development. IF2 does have some nonspecific DNA binding activity [27]. Thus, the enrichment of Mu end sequences with respect to Mu center sequences by immunoprecipitation is the best indicator of preferred IF2 binding at or near Mu ends although the enrichment of Mu end sequences with respect to host DNA is also clear in this analysis.

To ensure that the anti-S tag antibody was specifically precipitating Mu DNA bound to S-IF2-2, we compared the co-precipitation of Mu DNA (35 min postinduction) in induced lysogens expressing S-IF2-2 and untagged IF2-2 (Figure 2B). When the IF2-2 had no S tag, no more Mu DNA was captured in the immunoprecipitation than in the no-antibody control.

The results indicate that not only truncated IF2-2/3 but also full-length IF2-1 bind at or near Mu ends upon induction of Mu development, corroborating the role IF2 plays *in vitro* in promoting initiation of Mu DNA replication by restart proteins. *In vitro*, IF2 makes way for the binding of PriA [5], which binds to forked DNA structures [29], [30] such as the Mu fork, and PriA subsequently displaces IF2 from Mu DNA. The ChIP analysis by itself can only indicate a preponderance of IF2 binding around the Mu ends and does not rule out the possibility that IF2 binds at nearby sites. Nevertheless, these results together with the role IF2 plays *in vitro* strongly suggest that there are IF2 molecules bound at the Mu fork during lytic development. The role played by IF2 in Mu replication raises the question whether IF2 function can regulate the engagement or activity of restart functions.

### A deletion mutant that cannot express the full-length IF2 is unable to grow in the presence of MMS

**Figure 3:**
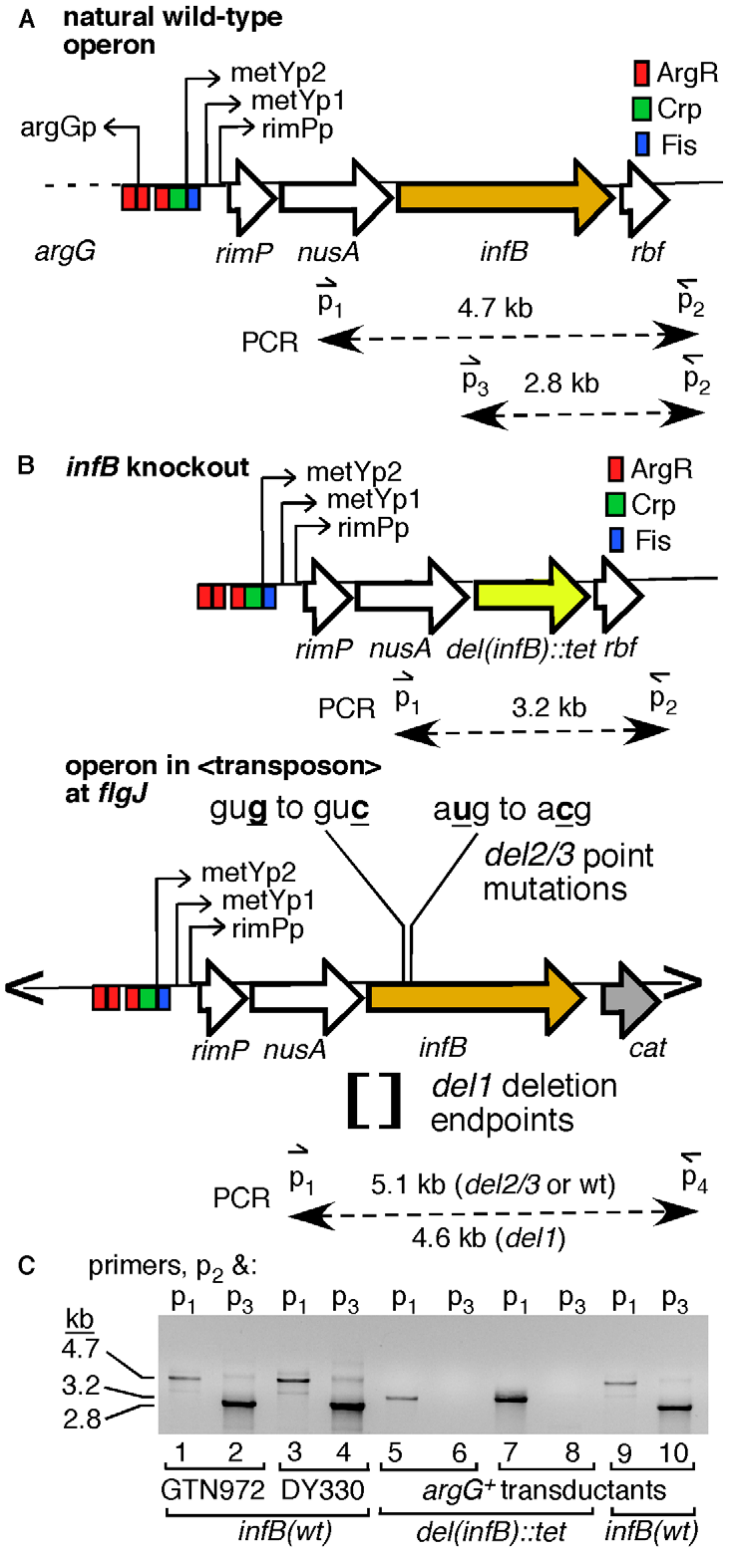
Introduction of a second single-copy *infB* allele and knockout of the natural allele. A) The natural *nusAinfB* operon. The operon is driven by the three indicated promoters, and the multiple cistrons are, starting from promoter proximal genes, *metY*, *rimP*, *nusA*, *infB*, *rbfA*, *truB*, *rpsO*, and *pnp* (not all shown). PCR primers used to specifically amplify the natural *infB* and not other copies of *infB* introduced in the cell are shown. B) The *del(infB)1::tet* allele and *nusA infB* allele harbored on an EZ-Tn5 transposon. The knockout allele precisely excises the *infB* cistron and replaces it with the *tet* cistron. The essential *infB* function is provided in single-copy form in a transposon (“<” and “>” represents transposon ends) integrated in *flgJ*. The transposon encodes chloramphenicol resistance (*cat*), allowing easy transfer to other cells, and the *nusAinfB* operon that includes the three ArgR binding sites up to the stop codon for *infB*. C) PCR analysis to detect knockout of the natural *infB* allele. DY330 is the strain used to engineer the *del(infB)1::tet* allele; GTN972 (GTN932 *del(argG)781::kan*) is the recipient strain for P1vir transduction from DY330*del(infB)1::tet<infB>*. Three typical GTN932 ArgG^+^ transductants are shown, two that coinherited *del(infB)1::tet* and one that did not (*infB(wt)*). The *infB* alleles of all mutants were amplified using the locus-specific primers and sequenced for verification.

We constructed a series of strains with *infB* alleles that only allow expression of full-length IF2-1 or the truncated forms IF2-2/3 to examine their effect on restart functions. The *infB* alleles were introduced into the chromosome where a transposon vector was inserted, and then the natural *infB* allele was knocked out by introduction of the *del(infB)1::tet* allele, which precisely deletes the natural cistron for IF2 (Figure 3A–3B). To prevent the expression of IF2-1, we deleted sequences around the translation initiation start site for IF2-1. Sequences from 14 nucleotides upstream of the IF2-1 start codon to 32 nucleotides upstream of the IF2-2 start codon were deleted (Figure 3B); this is known to permit expression of the truncated IF2 forms while eliminating IF2-1 expression [21]. The resulting allele, denoted as *infB(del1)* to indicate that the deletion prevents expression of IF2-1, supports the synthesis of only IF2-2 and IF2-3. Expression of the truncated IF2 forms were prevented by changing the start codons of IF2-2 and IF2-3, gug to guc (g474c) and aug to acg (t494c); these mutations have previously been shown to eliminate expression of the truncated forms while leaving a functional IF2-1 [21]. We shall refer to this allele as *infB(del2/3)* to indicate that the mutations prevent expression of IF2-2 and IF2-3 even though *del2/3* is not a deletion mutation. The resulting *infB del1*, *del2/3*, and wild-type (*wt*) alleles were introduced into the transposon site as part of the *nusA infB* operon (<*nusAinfB*> to signify that this is encoded within the transposon).

**Figure 4:**
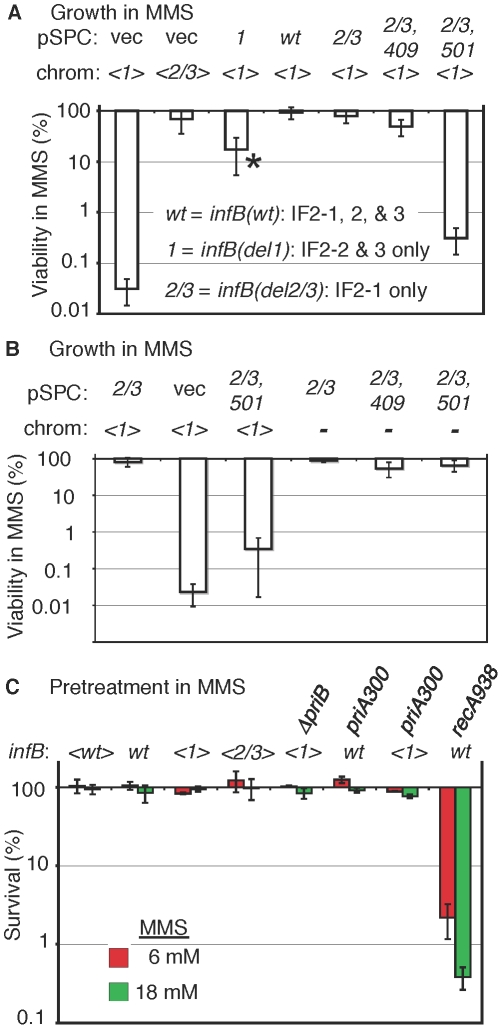
MMS sensitivity of the *<infB(del1)>* mutant and complementation by other *infB* alleles. A) GTN1114 *del(argA)743::kan* (GTN1156) and GTN1115 *del(argA)743::kan* (GTN1157) bearing pSPC*nusAinfB* plasmids that contain *infB(wt)*, *infB(del1)* (*1*), *infB(del1)* (*2/3*), or *infB(del2/3,D409E)* (*2/3,409*), or no *infB* (vec) allele. The asterisk (*) indicates that the strain required 40–42 h incubation (37°C) on MMS plates to yield sufficiently large colonies that could be counted, indicating especially slow growth on these plates (all other strains required 16–20 h incubation). The results are the average of 6–7 independent experiments. B) Multicopy *infB(del2/3,D501N)* allele can support growth in MMS provided that the chromosomal *<infB(del1)>* allele is not present. Viability of GTN932 *infB(del1) del(infB)1::tet* (GTN1114) and GTN932 *del(infB)1::tet* bearing the indicated pSPC*nusAinfB* plasmids (see the legend to panel A for the key) with and without MMS was determined (5–6 independent experiments). C) Sensitivity measured by 15-min exposure of cells at 6 mM and 18 mM MMS and plating in the absence of MMS (3–4 independent experiments). Strains with the indicated *infB* allele in the chromosome or transposon (*<>*) were used: GTN1050 (*<wt>*), GTN932 (*wt*), GTN1114 (*<1>*), GTN1115 (*<2/3>*), GTN1117 (*<1>* and *del(priB)302*), GTN381 (*wt* and *priA300*), GTN1323 (*<1>* and *priA300*), and GTN1376 (*wt* and *recA938*).

The natural *infB* allele could be readily knocked out by introducing the *del(infB)1::tet* allele when the operon in the transposon had *infB(wt)*, *infB(del1)*, and *infB(del2/3)* alleles. (The procedures for verifying deletion of the natural infB allele as illustrated in Figure 3C and for verifying *infB* alleles by PCR and sequencing will be described under Materials and Methods.) The <*infB(del2/3)*> and especially the <*infB(del1)*> strains display some measure of cold sensitivity, growing very slowly at 25°C and below, consistent with previous reports about strains with analogous alleles [21]. We determined that the strain with the single copy <*infB(del1)*> as sole allele was highly sensitive to MMS whereas the strains with <*infB(wt)*> and <*infB(del2/3)*> as sole alleles were not (Figure 4A and Figure S1A).

The results indicate that the *del1* mutation causes the inability to grow in the presence of MMS. The question is whether this is due to a general deficiency in repair, recombination, and restart functions, resulting from a generally deficient translation initiation function, or whether there is any specificity of the defect. We should note that the <*infB(del1)*> strain (ArgA^−^) was at least moderately proficient in homologous recombination measured by P1 transduction, although the frequency of Arg^+^ transductants was reduced approximately 5 fold compared to <*infB(wt)*> and <*infB(del2/3)*> strains (Figure S1B). While some reduction in homologous recombination frequency may be part of the phenotype of this strain, the reduction seen here is modest compared to the 20–50 fold reduction in P1 transduction demonstrated for the *priA* knockout strain [31].

### The full-length IF2-1 is necessary for growth in the presence of MMS

To determine whether it is indeed IF2-1 that is needed to maintain MMS-resistance, we complemented the <*infB(del1)*> allele of strain GTN1156 with the *infB(del2/3)* allele, harbored as part of a *nusA infB* operon on a plasmid with a pSC101 replicon, pSPC*nusAinfB(del2/3*). While the empty plasmid vector could not confer MMS-resistance and homologous recombination proficiency, IF2-1 expressed from pSPC*nusAinfB(del2/3)* did restore high viability on MMS plates (Figure 4A). In contrast, IF2-2/3 expressed from the plasmid-borne *infB(del1)* allele only partially restored viability on MMS plates (Figure 4A). While the multicopy *infB(del1)* allele did increase dramatically the viable count on MMS plates, the colonies grew up very slowly, and the viable count on these plates was still 5–10 fold lower than that of the strain with the multicopy *infB(del2/3)* allele (Figure 4A). These results illustrate functional differences between IF2-1 and IF2-2/3 in promoting recovery after MMS treatment. They also indicate that IF2-2/3 when expressed from a multicopy vector may compensate for the lack of IF2-1, albeit inefficiently.

To confirm that it was not just the DNA segment deleted in the *infB(del1)* allele but the full-length IF2-1 protein that was needed for complementation, we introduced IF2 G domain mutations, *infB(c1227a)* or *(c1501a)*, which result in the IF2 D409E and D501N alterations, respectively, into pSPC*nusAinfB(del2/3)*. The *infB(D409E)* allele is an example of a viable G mutant that is functional at 37°C [32] whereas *infB(D501N)* is a recessive allele that is lethal as a single-copy gene [33]. Introduction of pSPC*nusAinfB(del2/3,D409E)* into GTN1156, but not pSPC*nusAinfB(del2/3,D501N)*, restored MMS-resistance (Figure 4A). IF2-1 must therefore be providing the function needed for viability in MMS. The level of homologous recombination in the *<infB(del1)>* mutant, examined by P1 transduction, could also be increased by supplying the various *infB* alleles on the plasmid vector (Figure S1C). Due to the relatively modest effect on homologous recombination, this aspect of the *infB(del1)* mutant was not further examined.

### Dominant negative effect of the *<infB(del1)>* allele over multicopy *infB(del2/3,D501N)*

The *<infB(del1)>* strain, which produces only IF2-2/3 and has extremely low viability in the presence of 6 mM MMS, attains high viability when complemented with the plasmid-borne *infB(del2/3)* allele, which restores IF2-1 production (Figure 4A and 4B). This indicates that the multicopy *infB(del2/3)* allele is dominant over the *<infB(del1)>* allele. The inability of pSPC*nusAinfB(del2/3,D501N)* to restore efficient growth of the *<infB(del1)>* strain in MMS could indicate the inactivation of a necessary function of IF2-1 by the D501N mutation. Alternatively, the <*infB(del1)*> allele may be dominant negative over the multicopy *infB(del2/3,D501N)* allele in terms of supporting growth in MMS.

Although the D501N mutation is lethal when present as a single-copy *infB* allele, this mutation is recessive to the wild-type allele [33]. We therefore tested whether the multicopy *infB(del2/3,D501N)* allele on the plasmid could support viability by itself. The natural *infB* allele in strain GTN932 that bears plasmids pSPC*nusAinfB(del2/3)*, pSPC*nusAinfB(del2/3,D409E)*, or pSPC*nusAinfB(del2/3, D501N)* could readily be knocked out, leaving the *infB* on the plasmid as the sole allele in the cell. This allowed us to test whether or not IF2-1(D501N), expressed from multicopy *<infB(del2/3, D501N)>*, is defective in a function that IF2-1 provides but IF2-2/3 fails to perform. Although the strain with the multicopy *infB(del2/3,D501N)* as the sole allele grew relatively slowly, requiring at least twice the incubation time as the other two strains for growth, it was clearly viable and also retained significant viability on MMS plates, comparable to viability of analogous strains with *infB(del2/3)* and *infB(del2/3,D409E)* as sole alleles (Figure 4B). That is, the multicopy *infB(del2/3,D501N)* is able to support high viability in MMS so long as the *<infB(del1)>* allele is absent. Introduction of the D501N mutation to the multicopy *infB(del2/3)* allele thus results in loss of dominance over *<infB(del1)>*, not in the loss of a function needed to maintain viability in MMS. These results suggest that IF2-2/3, at levels produced from the single-copy *<infB(del1)>* allele, is performing a function in a way that aggravates problems which the cells encounter during growth in MMS, outcompeting IF2-1(D501N) that is able to carry out the function appropriately to maintain viability. In other words, IF2-2/3 does not necessarily lack the capacity to perform the IF2-1 function. Rather, it appears to carry it out in a way that dramatically reduces viability. That is, the recessive properties of *infB(D501N)* with respect to the *infB(del1)* allele, including its ability to support resistance to MMS as the sole multicopy allele, suggest that MMS sensitivity of the *<infB(del1)>* strain is not simply due to a general deficiency in translation initiation function when only IF2-2/3 is present.

### Characteristics of MMS sensitivity of the *<infB(del1)>* mutant, an attribute shared by the *priA300* mutant

**Figure 5:**
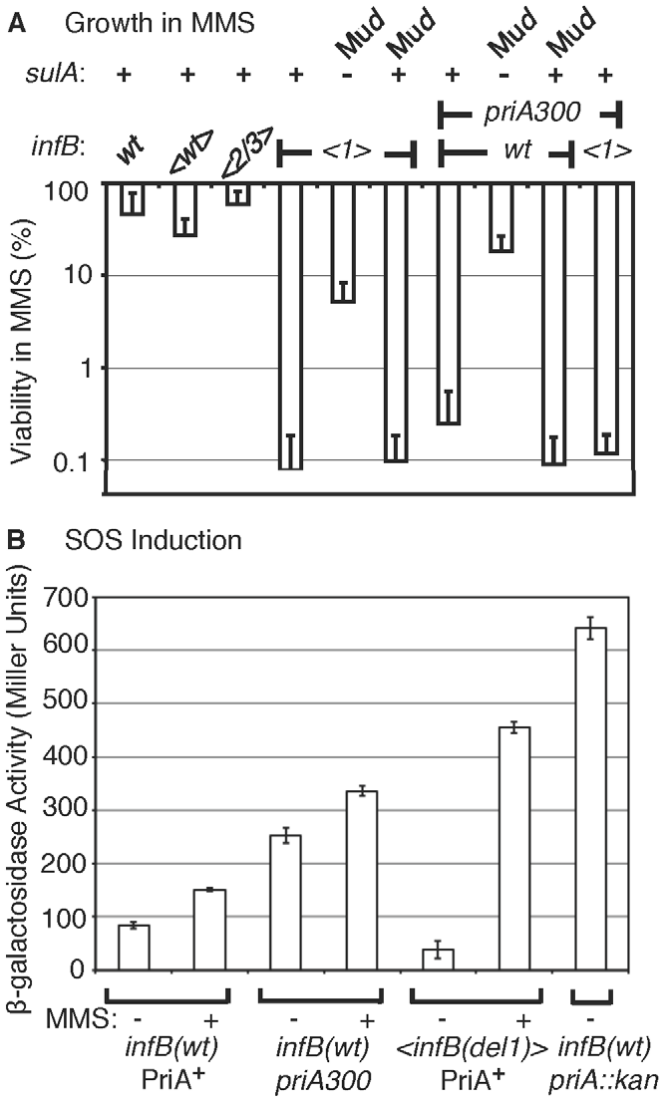
Characteristics of MMS sensitivity exhibited by *<infB(del1)>* mutants and similarity to *priA300*. A) Sensitivity of *<infB(del1)>* and *priA300* mutants for growth in MMS and suppression by a *sulA* mutation. The indicated strains are the same as those listed in the legend to Figure 4C. In addition, the *sulA::*Mu*d(lac,Ap,B::Tn9)* allele is present in GTN1387 (*<infB(del1)>* SulA**^−^**) and GTN1384 (*priA300* SulA**^−^**), and the Mu*d(lac,Ap,B::Tn9)* is integrated in a site other than *sulA* in GTN1399 (*infB(del1*) SulA**^+^**) and GTN1396 (*priA300* SulA**^+^**). Viability of GTN1376 (*recA938*) on MMS plates was less than 10**^−^** ^5^%. Results are the average of at least 3 independent determinations. B) SOS induction monitored using the *sulA::lacZ* reporter. GTN1385 (*infB(wt)* PriA^+^), GTN1384 (*priA300*), and GTN1387 (*<infB(del1)* PriA^+^) were grown in LB to OD_600_ of 0.3. To 2-ml portions of each culture, MMS was added to 18 mM final concentration. β-galactosidase activity in MMS-treated (+) and untreated (−) cultures was measured. β-galactosidase activity of untreated GTN1639 (GTN1385 *priA2::kan*) is shown for comparison.

We next determined whether the MMS sensitivity of the *<infB(del1)>* mutant reflected deficiency in the levels of repair or restart proteins in these mutants. In the analysis described above, MMS resistance was measured by growth of cells on plates containing MMS. By this analysis, cells must not only survive initial exposure to the DNA-damaging agent but also grow into colonies in its presence. We also measured the ability of strains exposed to MMS to recover and grow in the absence of MMS in order to assess their capacity to repair DNA lesions and restart DNA replication. Strains that are defective in genes such as *priA*, *recA*, and *polA* that participate in DNA repair or replication restart are known to be quite sensitive as measured by initial exposure for 15 min in MMS and plating without MMS to determine the number of survivors; the *alkA tag* mutant, which is defective in a major mechanism for repairing alkylated bases (base excision repair), is also sensitive to MMS by this criteria [34]. The <*infB(del1)*> mutant was as resistant to MMS as *infB(wt)* strains by this criteria (Figure 4C), with MMS resistance comparable to strains with natural *infB*, <*infB(wt)*>, and <*infB(del2/3)*> alleles; in contrast the *recA938* mutant was highly sensitive by this criteria (Figure 4C). It should be noted that when cells were deficient in both the PriA-PriB and PriA-PriC pathway (deficient in both PriB and PriC), they had very low viability even without MMS treatment (Figure S2A). As restart mutants tend to have very low viability even without MMS, we measured MMS sensitivity of a *del(dnaT)759::kan* mutant with a *dnaC(a491t)* suppressor mutation, which greatly increases cell viability. Even with the suppressor mutation, the *dnaT* knockout strain was significantly more sensitive to the 15-min MMS treatment (Figure S2B) than the *<infB(del1)>* mutant. These results indicate that levels of repair and restart factors in the <*infB(del1)*> strain are sufficient for the recovery of DNA replication and cell growth after DNA damage by MMS. However, there was a 1000-fold reduction in viability of the <*infB(del1)*> mutant on 6 mM MMS plates (Figure 5A). That is, the <*infB(del1)*> strain is proficient in repairing DNA damage and resuming DNA replication after the 15-min exposure in MMS, but it is severely defective in its ability to sustain growth in MMS. Thus, the *<infB(del1)>* mutant is not able to cope with the sustained damage to DNA during cell growth. This could indicate that a repair or restart factor, although not deficient, is sufficiently low such that it cannot keep up with constant DNA damage inflicted on MMS plates; alternatively, it is possible that the regulation of repair and restart processes are not appropriate for efficiently supporting DNA replication under these conditions.

Introduction of the *sulA::*Mu*d(lac, Ap, B::Tn9)* allele greatly restored viability of the *<infB(del1)>* mutant in MMS (Figure 5A). The *sulA* gene, which is a component of the SOS system induced by DNA damage, is a cell division inhibitor [35]. In mutants such as the *priA* null strain, which has a constitutively induced SOS system, the high expression of *sulA* results in loss of viability, which can be largely restored by *sulA* mutations [36]. It is important to note that the *sulA::*Mu*d(lac, Ap, B::Tn9)* allele did not fully restore viability to the *<infB(del1)>* mutant. Moreover, scorable colonies on MMS plates required incubation for over 36 hours at 37°C whereas *infB(wt)* colonies readily arose in 16 hours. That is, the *sulA* mutation did not fully revert *<infB(del1)>* to the wild-type phenotype.

Interestingly, the *priA300* mutant had a phenotype much like the <*infB(del1)*> mutant, resistant to MMS when exposed to MMS and plated in its absence but highly sensitive when plated on 6 mM MMS plates (cf. Figure 4C with Figure 5A). The *priA300* allele encodes for a helicase-deficient PriA that is fully proficient in primosome and replisome assembly by the PriA-PriB pathway [19], [37]. The *priA300* mutant has previously been shown to have essentially a wild-type phenotype unless that mutation is combined with mutations affecting other restart functions such as *priB*; wild-type properties of the *priA300* mutant include homologous recombination proficiency and relatively high UV resistance [19], [38]. As with the *<infB(de11)>* mutant, the *sulA::Mud(lac,Ap,B::Tn9)* allele could restore viability of the *priA300* mutant in MMS (Figure 5A). In addition, *priA300* was epistatic with the *infB(del1)* allele, causing no significant increase in MMS sensitivity (Figure 4C and Figure 5A). In contrast, the *<infB(del1)> del(priB)302* combination (GTN1117) was synergistic, reducing viability to 0.010±0.002% on the MMS plates. The *priB* knockout alone did not have such a severe effect; the *<infB(wt)> del(priB)302* strain (GTN1133) had a viability of 43±8% on MMS plates. In addition, knockout of *priC* did not increase UV sensitivity; the *<infB(wt)> del(priC)752::kan* strain (GTN1059) had a viability of 84±7% on MMS plates. These results indicate that the PriA-PriC pathway, which requires PriA helicase, is not solely responsible for allowing cell growth in the presence of MMS and that the PriA-PriB pathway most likely makes a significant contribution to mechanisms dependent on PriA helicase as well. We shall further examine the interactions of *priA300* and *del(priB)302* with the *<infB(de11)>* and *<infB(de12/3)>* alleles by UV sensitivity. The epistatic relationship between the *infB(del1)* and *priA300* alleles suggests that the loss of IF2-1 specifically affects the activity or engagement of factors in restart pathways that require PriA helicase.

Despite the high MMS sensitivity of the *<infB(del1)>* strain, it did not resemble the *priA* knockout mutant in terms of having constitutively high levels of SOS induction (Figure 5B). Expression from the *sulA::lacZ* SOS reporter was significantly lower than the strain with wild-type *priA* and *infB* and the *priA300* strain. The latter strain had moderate basal levels of SOS induction, which was significantly less than that of the *priA* knockout. Treatment of the wild-type and *priA300* strains with 18 mM MMS elicited moderate increases in SOS expression; in contrast, treatment of the *<infB(del1)>* strain elicited over a 10-fold increase in SOS expression, consistent with the role of SOS induction reducing the strain's viability upon MMS treatment,

### UV sensitivity of *<infB(wt)>*, *<infB(del1)>*, and *<infB(del2/3)>* strains and epistasis analysis with restart functions

**Figure 6:**
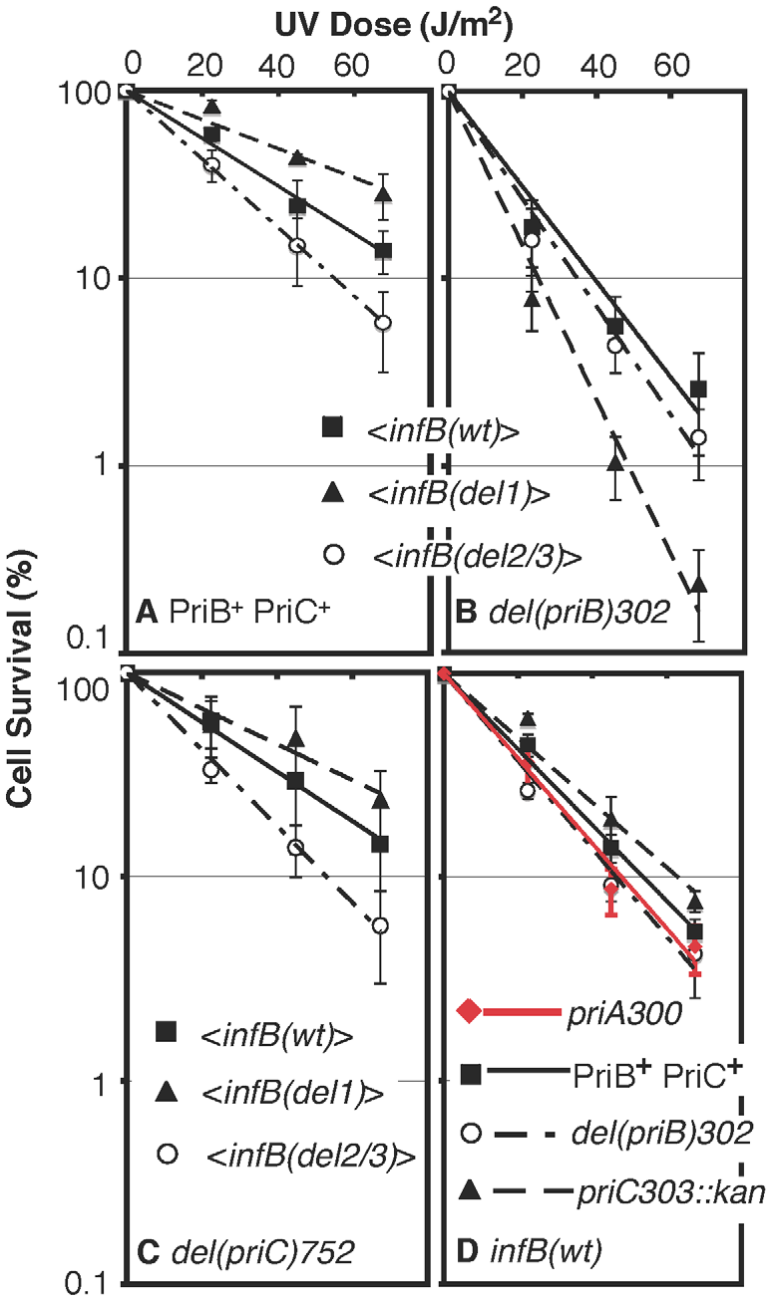
UV sensitivity of *<infB(del1)> and <infB(del2/3)>* and epistasis analysis with *priB* and *priC*. A) UV sensitivity of *<infB(wt)>* (GTN1050), *<infB(del1)>* (GTN1114), and *<infB(del2/3)>* (GTN1115) mutants. B) UV sensitivity of mutants with *del(priB)302* and the *<infB(wt)>* (GTN1133), *<infB(del1)>* (GTN1117), or *<infB(del2/3)>* (GTN1119) alleles. C) UV sensitivity of mutants with *del(priC)752* and the *<infB(wt)>* (GTN1059), *<infB(del1)>* (GTN1135), or *<infB(del2/3)>* (GTN1137) alleles. D) As reference, the effect of *priA300* (GTN381), del*(priB)302* (GTN394) and *priC303::kan* (GTN387) alleles in the GTN932 genetic background was examined. The *priB* and *priC* knockout alleles were the first knockout mutations of these genes to be characterized [39]. All results are the average of at least 3 independent determinations.

Although the *<infB(del1)>* strain was sensitive to growth in MMS, it was slightly more resistant to UV light than the *<infB(wt)>* strain (Figure 6A). In fact, the *<infB(del2/3)>* mutant, which was found to be the most MMS-resistant, was slightly more UV sensitive than the *<infB(wt)>* strain (Figure 6A). These results do not rule out the possibility that the *del1* and *del2/3* mutations impair or knock out restart mechanisms engaged after UV irradiation. As there are multiple restart pathways in the cell, the PriA-PriB and PriA-PriC pathways being the two major ones [18], the *del1* or *del2/3* mutation may predominantly affect only one pathway but not the other. To test this possibility, we examined the effect of the *infB* alleles in combination with *priB* or *priC* knockout alleles.

**Figure 7:**
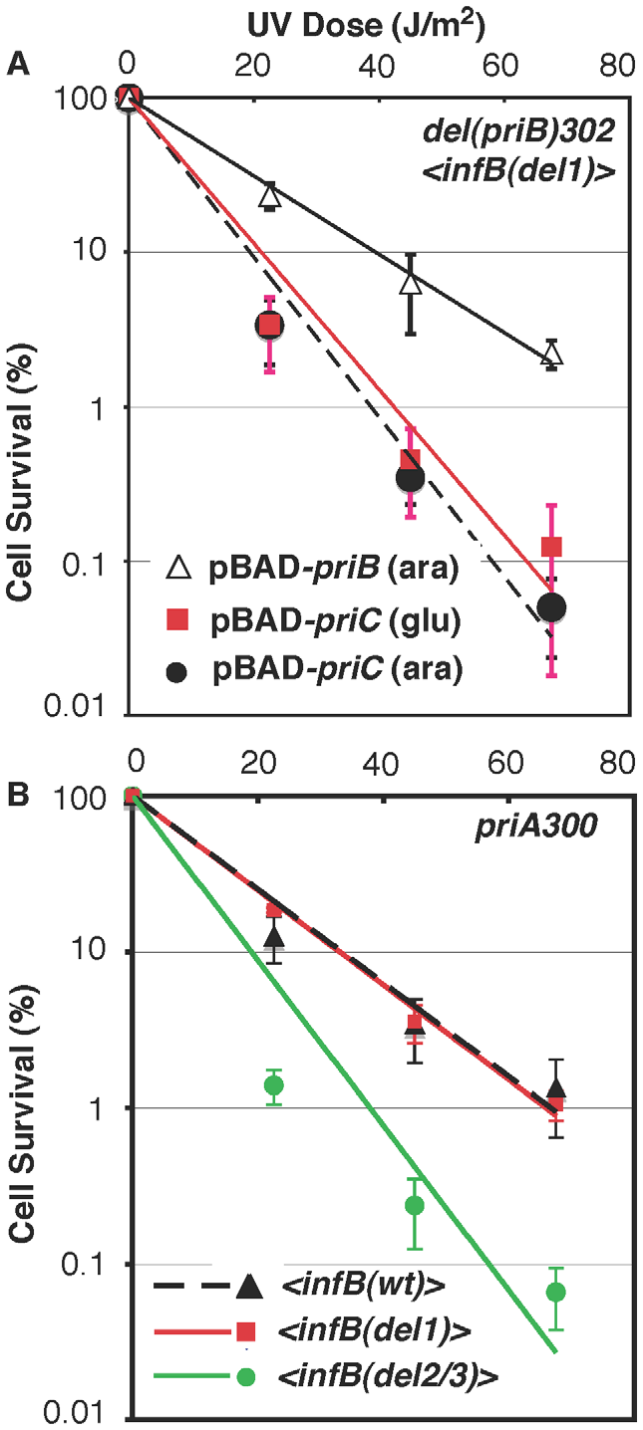
Epistasis analysis of *infB* alleles with *del(priB)302* and *priA300*. A) UV sensitivity of GTN1117 expressing *priB* or *priC* from pBAD24. GTN1117, which is *del(priB)302 <infB(del1)>*, bearing plasmid pBAD24-*priB* or pBAD24-*priC* were grown up in LB medium containing 100 µg/ml ampicillin and 0.02% L-arabinose or 0.2% D-glucose as indicated. After UV irradiation, cells were plated on 0.02% arabinose/LB and plain LB plates for viability, both of which produced identical results. B) Interaction of *infB* alleles with *priA300*. Survival of *priA300* strains with the *<infB(wt)>* (GTN1298), *<infB(del1)>* (GTN1323), and *<infB(del2/3)>* (GTN1297) after UV irradiation was measured.

It is well established that the knockout of *priB* or *priC* has little to no effect by itself [39] in contrast to the *priA* or *dnaT* knockouts, which affects both major restart pathways and elicits high sensitivity to DNA-damaging agents and low viability [18], [36], [40]. As expected, neither the *priB* nor *priC* knockout had any effect on UV sensitivity when introduced into the parent strain (GTN932) used to construct the various *<infB>* mutants (Figure 6D). While the *del(priC)752* allele had absolutely no effect on single-copy *<infB(wt)>*, *<infB(del1)>*, and *<infB(del2/3)>* strains (Figure 6C; cf. with Figure 6A), the *del(priB)302* clearly had a synergistic effect with the *infB(del1)* mutation to elicit relatively high UV sensitivity (Figure 6B). This finding that the *priB* knockout, but not the *priC* knockout, is synergistic with the *<infB(del1)>* allele to increase UV sensitivity indicates that the loss of full-length IF2-1 diminishes the PriA-PriC pathway for recovery after UV irradiation. Introduction of a pBAD24-*priB* plasmid into the *del(priB)302 <infB(del1)>* strain (GTN1117), allowing the expression of PriB driven by the P_BAD_ promoter with arabinose as inducer, increased its UV resistance to levels comparable to the *del(priB)302 <infB(wt)>* strain (GTN1133; Figure 7A), confirming that the deficiency of GTN1117 can be reversed by expressing PriB. This indicates that the activity of repair and restart proteins needed for recovery after UV irradiation in GTN1117, which has the <*infB(del1)*> allele, is comparable to that in GTN1133, which has the <*infB(wt)*> allele. Therefore, the increased UV sensitivity of GTN1117 with respect to GTN1133 is most likely due to some type of deficiency in the PriC-dependent pathway. We were unable to measurably increase UV resistance by expressing PriC from pBAD24-*priC* (Figure 7A). Indeed, PriC in its active form must be present in GTN1117. When the chromosomal *priC* was knocked out in pBAD24-*priC*/GTN1117 (GTN1566), expression of PriC from the plasmid vector became essential for viability with or without pre-treatment with MMS (Figure S2A), viability being less than 0.1% in the presence of glucose. In the presence of arabinose, viability of GTN1566 with or without MMS treatment was comparable to the strain with an intact chromosomal *priC*. That is, active PriC can be expressed from pBAD24-*priC* or the chromosomal *priC* gene in the *<infB(del1)>* genetic background, and supplementation of PriC expression in GTN1117 from the plasmid cannot restore any measure of UV resistance. These results suggest that its relatively high UV sensitivity is not caused by a deficiency in PriC, PriA, and DnaT.

Although the *del(priB)302 <infB(del1)>* strain (GTN1117) has high UV sensitivity, its ability to recover after a 15-min exposure to MMS was comparable to the wild-type control (Figure 4C). Moreover, Mu plating efficiency on this strain is not dramatically reduced, indicating that the PriA-PriC pathway can promote Mu replication in the absence of IF2-1 (Figure S3), a result consistent with properties of Mu replication *in vitro* [5]. In general, the Mu plating efficiencies on the various <*infB(wt, del1, or del2/3)*> strains, whether in the PriB^+^PriC^+^, *del(priB)302*, or *del(priC)752::kan* genetic backgrounds, were nearly the same. These results indicate that restart proteins needed to promote Mu replication by the PriA-PriC pathway are present at sufficient levels to support lytic development. They also suggest that the defect of the *infB(del1)* allele is not a deficiency in restart activity needed for recovery but rather in the regulation of restart activity needed to maintain replication in the presence of the DNA-damaging agent.

Although the effect is not as much as in the *<infB(del1)>* background, the *del(priB)302* allele also did significantly increase UV sensitivity when introduced into the *<infB(wt)>* background (*cf.* the solid square data points in Figure 6A and 6B) whereas it had essentially no effect in the natural *infB(wt)* background (Figure 6D). This may reflect a small change in relative or absolute levels of full-length and truncated IF2 when the *infB* allele is expressed from the transposon site, a change that has no discernible effect unless specific restart mechanisms are inactivated as with the *del(priB)302* mutation. Interestingly, in the *<infB(wt)>* background both the *priA300* (Figure 7B) and the *del(priB)302* (Figure 6B) allele increased UV sensitivity to the same level. Like the *del(priB)302*, the *priA300* allele is known to have little effect on UV sensitivity [19], and indeed we found essentially no effect of the *priA300* allele in the GTN932 background (Figure 6D), which has the natural *infB(wt)* allele. As we described above, the *priA300* and *<infB(del1)>* alleles both independently elicit sensitivity to growth in MMS, and the two mutations are epistatic for this trait, consistent with a model in which PriA helicase and IF2-1 function in the same pathway to maintain efficient growth in MMS. In the UV sensitivity analysis, the *infB(del1)* allele was also found to be epistatic with *priA300*, not being able to elicit further UV sensitivity in the *priA300* background (Figure 7B). That is, loss of IF2-1 attenuates pathways dependent on PriA helicase such as the PriA-PriC pathway. In contrast, the *infB(del2/3)* allele was synergistic with *priA300* to increase UV sensitivity (Figure 7B). The results indicate that loss of IF2-2/3 from the *infB(del2/3)* allele results in deficiency of a restart pathway that is distinct from the IF2-1/PriA helicase-dependent pathway. Mu plating efficiency on the three *priA300* strains with the *<infB(wt)>*, *<infB(del1)>*, and *<infB(del2/3)>* were essentially the same, the titer obtained on the latter two strains being greater than 90% of the titer on the *priA300 <infB(wt)>* strain. Thus, as with the *del(priB)302 <infB(del1)>* combination, which also synergistically contributes to high UV sensitivity, the *priA300 <infB(del2/3)>* combination does not lead to an inability to initiate Mu replication by the available host restart machinery.

### Sporadic SOS induction of the *del(priB)302 <infB(del1)>* mutant

What is notable about the UV sensitivity analysis is that the combination of *priA300 <infB(del2/3)>* or *del(priB)302 <infB(del1)>* mutations does not produce the extremely severe phenotype of the *priA300 del(priB)302* combination, which elicits a phenotype analogous to the *priA* knockout [19]. That is, loss of IF2-1 or IF2-2/3 does not result in the inability to promote replication restart by the respective pathways they influence, but rather the loss of each IF2 isoform affects some mechanism needed to maintain high viability when the restart mechanism is engaged after DNA damage. However, under normal growth conditions or if cells are allowed to recover after MMS treatment or UV irradiation without the presence of DNA damaging agents, there is little effect of knocking out IF2 isoforms, and a mild effect is seen when these mutations are combined with the restart mutation *del(priB)302* or *priA300*, which by itself has little effect under normal growth conditions. We examined the cell morphology of the various *infB* mutants to examine whether there is an increased incidence of sporadic SOS induction, leading to filamentation [41] of a small fraction of the cells in the population.

The strains with the single *del(priB)302* or *<infB(del2/3)>* mutant had essentially wild-type morphology (Figure S4A), 100% of cells being 0–8 µm in length when at least 40,000 cells were analyzed. Cells with the single *<infB(del1)>* or *priA300* mutation (GTN1114 and GTN1298, respectively) tended to be longer in size, with a higher incidence of moderate sized filaments (examples of moderate filaments are indicated by white arrows). In a sample of 1000 cells, 1% of the cells were in the 8–30 µm range for GTN1114 and GTN1298. Consistent with the relatively low basal levels of SOS expression measured for the *<infB(del1)>* mutant at the macroscopic level (Figure 5B), the level of its filamentation was quite low compared with that of the *priA* knockout mutant (Figure S4B), but the moderate filamentation suggests an increased incidence of sporadic SOS induction.

What was notable for the synergistic *<infB(del1)> del(priB)302* combination (GTN1117) was that it gave rise to a low but significant frequency of very large filaments greater than 30 µm (Figure S4A). The incidence of filaments over 30 µm in size was found to be 0.13% in a screening of 33,000 total cells, most of these large filaments (0.10% of total cells) being over 50 µm in length. Only one other combination of an *infB* allele with the *priA300*, *del(priB)302*, or wild-type restart functions (Figure S4A) yielded any filaments over 50 µm in 100,000 cells screened. The mutant with the synergistic *<infB(del2/3)> priA300* combination (GTN1297) produced filaments greater than 30 µm at a significantly lower frequency of 0.02% in a screening of 100,000 cells, of which only 3 were greater than 50 µm. Filaments in the 30–50 µm range also arose with the single *<infB(del1)>* or *priA300* mutants (GTN1114 and GTN1298, respectively) but with a frequency of no more than one in 40,000 cells. No filaments of greater than 30 µm were detected with the *<infB(wt)>* (GTN1050), *<infB(del2/3)>* (GTN1115), *del(priB)302* (GTN1133), and the *<infB(del1)> priA300* (GTN1323) strains when at least 100,000 cells were examined. The results indicate that the *<infB(del1)> del(priB)302* mutant (and, to a lesser extent, the *<infB(del2/3)> priA300* mutant) has an increased incidence of very high SOS induction (leading to the formation of giant filaments) in a small fraction of the cell population growing in LB, suggesting a reduced capacity to cope with accidents that might occur during DNA replication for normal cell growth. However, these mutants clearly do not have the characteristics of extensive SOS induction as with a *priA* knockout strain such as GTN430 (Figure S4B; 3% of cells producing filaments greater than 30 µm in a sample of 4000 cells, 2% greater than 50 µm).

### Characteristics of a restart mutant with a suppressor mutation in *dnaC*

The characteristics of strains such as the *<infB(del1)>* or *priA300* mutant are more akin to a *priA* knockout strain that has acquired a suppressor mutation in *dnaC* (Table 1). GTN412, which is a Mu*cts62* lysogen, can support Mu replication upon thermoinduction to yield a high level of infective centers, has a high level of viability on MMS plates, and has a relatively low level of expression from its SOS reporter gene (*dinD::lacZ*). Introduction of the *priA* knockout decreased viability on MMS and formation of Mu infective centers by several orders of magnitude. The presence of a suppressor mutation in *dnaC* (GTN522) did diminish cell filamentation (Figure S4B; the number of filaments >30 µm are reduced to 0.05% from 3%, measured in a sample of 15,000 cells), reduce the level of SOS induction as indicated by the *dinD::lacZ* reporter, and restore the ability to form Mu infective centers, but this strain retained the severe sensitivity to growth in the presence of MMS, a central feature of the both the *<infB(del1)>* and *priA300* mutants. This is consistent with the ability of the *dnaC* suppressor mutation to bypass the requirement for PriA to initiate DNA synthesis at forked DNA structures [38], [42]; however, without PriA the mechanism for promoting replication restart and promoting high viability in the presence of MMS (the IF2-1/PriA helicase-dependent pathway) appears to be compromised. In the same way, a *priA300* or *<infB(del1)>* mutant may be able to promote replication restart by a less preferred pathway, which may permit replication restart to proceed but does not do so in a way that supports high viability during growth in the presence of MMS.

**Table 1:** Characteristics of a *priA* knockout mutant with a *dnaC* suppressor.

Strain	Genotype	MMS resistance^a^ *(% survival)*	SOS^b^ *(units)*	Mu Infective Centers^c^ *(% forming plaques)*
GTN412	basic^d^	36±2	42±1	23±3
GTN430	*priA2::kan*	<0.005	216±6	<0.08
GTN522	*priA2::kan sup* e	<0.005	65±1	16±3

a Cells were plated on LB plates containing 6 mM MMS. Cells were grown at 30°C.

b The amount of SOS induction was measured using the *dinD-lacZ* fusion present in each strain. The level of β-galactosidase activity is expressed as Miller units, which are a measure of enzymatic activity per cell density.

c The portion of viable cells forming infective centers when plated on a lawn of GTN932 indicator culture at 42°C was measured.

d GTN412 has the following basic genotype, which is shared by GTN430 and GTN522: *his::*Mu*c*ts62 *del(priB)302 dinD1::*Mu*d(lac,Ap) del(gpt-lac)5*.

e This is a suppressor mutation in *dnaC*, changing the gug codon for val-135 to aug (met).

## Discussion

### Conclusions

The present work indicates a special relationship between the PriA helicase function and IF2-1 (see Table 2). Both the PriA helicase function and IF2-1 are required to allow cells to grow with maximal viability in the presence of MMS. Nevertheless, neither of these mutants display the severe characteristics of the *priA* knockout, having UV resistance that is comparable to wild type and being able to recover from MMS treatment with very high viability provided that it can do so in the absence of MMS. The defect of the *priA300* mutant, previously shown to have nearly a wild-type phenotype [19], is a surprising new phenotype, being defective in the ability to grow in the presence of MMS but not in its ability to recover from MMS treatment. Even more surprising is the finding that the loss of the IF2-1 function elicits the same phenotype. Another characteristic which indicates that the *infB(del1)* allele affects some aspect of replication restart is the suppressing effect of knocking out *sulA*, a mutation that greatly increases viability of both the *infB(del1)* and *priA300* mutant on MMS plates. Moreover, MMS treatment of *inf(del1)* mutant promotes an especially high level of SOS induction compared to the level promoted in wild type.

**Table 2:** Comparison of Attributes of *priA300* and *infB(del1)* mutant.

*infB(del1)*	*priA300*
Very poor growth in 6 mM MMS, with approximately a 1000-fold reduction in viability.	
Efficient recovery after treatment with 6–18 mM MMS when allowed to recover in the absence of MMS	
The mutant allele causes no significant increase in UV sensitivity compared with the wild-type allele	
Viability during growth in MMS is greatly but not totally restored when the *sulA* gene is disrupted.	
Sensitivity to UV and loss of viability during growth in the presence of MMS is enhanced in combination with the *priB* knockout but not with the *priA300* allele.	Sensitivity to UV is enhanced in combination with the *infB(del2/3)* allele but not with *infB(del1)*.
Even in combination with the *priB* knockout, the phenotype is not as severe as the *priA* knockout mutant, with relatively mild UV sensitivity, the ability to recover efficiently after pretreatment with 18 mM MMS, and no extensive SOS induction except for increased sporadic filamentation.	In combination with the *priB* knockout, elicits a phenotype like the *priA* knockout, including extreme sensitivity to DNA damaging agents, very low viability, and a persistent and high level of SOS induction resulting in extensive cell filamentation [19].

A relationship between full-length and truncated IF2 isoforms and replication restart functions is further indicated by UV sensitivity analysis. Both the *<infB(del1)>* and *<infB(del2/3)>* exhibit UV resistance comparable to wild type, but the combinations of *<infB(del1)> del(priB)302* and *<infB(del2/3)> priA300* significantly enhance UV sensitivity. Moreover, the *<infB(del1)> del(priB)302* mutant (and, to a lesser extent, the *<infB(del2/3)> priA300* mutant) display an increased frequency of sporadic SOS induction, indicated by the increased frequency of very long filaments over 30 µm. Clearly, the general population of these cells do not display the same high level of SOS induction of the *priA* knockout cells at the macroscopic level. The sporadic nature of filamentation is consistent with the thinking that these cells are mostly proficient in coping with accidents of DNA replication which may arise during normal growth conditions, unlike the *priA* knockout that copes with such accidents poorly. One would expect that only a small minority of cells would need to cope with a large number of DNA lesions during growth in LB unless a DNA-damaging agent such as MMS is present. The combination of *<infB(del1)> del(priB)302* and *<infB(del2/3)> priA300* alleles may sufficiently attenuate the major pathways that lead from DNA damage to replication restart, thus manifesting a modest but significant increase in sensitivity to UV irradiation.

**Figure 8:**
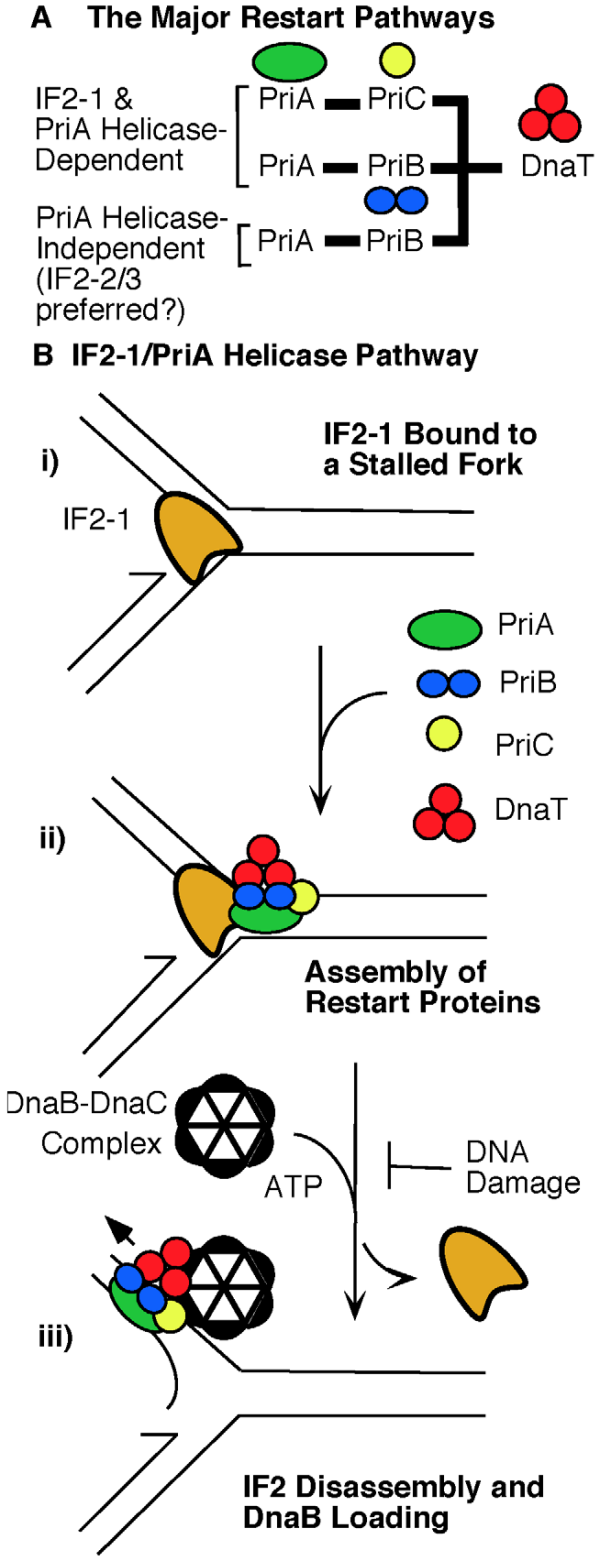
Role of IF2 isoforms in the major replication restart pathways. A) The major restart pathways and the influence of IF2 isoforms. Genetic analysis indicates that IF2-1 influences the restart pathways dependent on PriA helicase, including not only PriA-PriC pathway but also part of the PriA-PriB pathway. IF2-2/3 may play a prominent role in the pathways that do not require PriA helicase. The diagram should not be interpreted to indicate that IF2-2/3 cannot participate in reactions involving PriA helicase or that IF2-1 cannot participate in reactions where PriA's helicase is inactive. However, under these circumstances restart pathways do not function optimally to maintain maximal cell viability. The minor, less robust pathways such as the Rep-PriC pathway are not shown here. These PriA-independent pathways require suppressor mutations in *dnaC* to support a significant level of cell viability [18], [38], [39]. B) Model for the IF2-2/PriA helicase pathway. i) The starting point is a stalled replication fork with bound IF2-1. ii) Binding of restart proteins. The key protein to bind at this stage is PriA, which is poised to displace IF2-1 upon activation of its helicase activity. iii) Replisome assembly. PriA helicase action disassembles IF2-1 from the template, making way for initiation of DNA replication. It is hypothesized that removal of IF2-1 by PriA helicase can be regulated, extensive damage to the DNA template being able to inhibit this process and prevent replication restart. Removal of IF2-2/3 by PriA helicase bypasses this regulation, and IF2-2/3 may also be removed from the DNA by other mechanisms.

The epistatic relationship between the *priA300* and *infB(del1)* alleles revealed by both UV sensitivity and viability on MMS plates indicates that IF2-1 and PriA helicase function in common pathways as proposed in Figure 8A. This includes mechanisms in both the PriA-PriB and PriA-PriC pathway, for neither the *priB* or *priC* knockout has the severe effect of *priA300* for growth on MMS plates. What remains of the major restart pathways when PriA helicase is inactive are mechanisms in the PriA-PriB pathway that can operate in the *priA300* background [19]. Thus, the effect of the *infB(del2/3)* allele in this genetic background (increased UV sensitivity and increased incidence of sporadic cell filamentation) suggests that IF2-2/3 plays a role in this pathway. However, we have yet to find a phenotype for the *infB(del2/3)* allele alone, comparable to MMS sensitivity of the *infB(del1)* mutant, and whether IF2-2/3 is a key participant in PriA helicase-independent restart mechanisms (Figure 8A) remains be determined.

Finally, the characteristics of the <*infB(del1)*> and *priA300* mutants and especially the *infB(del1) del(priB)302* double mutant are more like the *priA* knockout with a suppressor mutation in *dnaC* rather than the *priA* knockout with no suppressor. The <*infB(del1)*> and *priA300* mutants, like the *priA* knockout with suppressor, do not exhibit the extreme sensitivity to UV irradiation, the massive cell filamentation, and the inability to support Mu replication that is characteristic of the *priA* knockout. Nevertheless, all of these mutants are not able to grow efficiently on media containing 6 mM MMS, their viability on MMS plates being approximately 0.1% or less. For the *priA* knockout, the *dnaC* suppressor allows replication restart to proceed, but the bypass of the restart proteins compromises maintenance of high cell viability when DNA replication proceeds during relatively high rates of DNA damage. Similarly, replication restart mechanisms can still operate in the *<infB(del1)>* mutant, and the lack of IF2-1 may bypass the preferred pathway that maintains high cell viability during growth in the presence of MMS. As IF2-1 and IF2-2/3 share 726 common residues, IF2-1 having 157–164 extra residues at the N-terminal end, it is quite conceivable that IF2-2/3 can replace IF2-1 in the IF2-1/PriA helicase-dependent pathway, allowing replication restart to proceed but lacking the function need to maintain high cell viability. The ability to grow under conditions that damage DNA at elevated levels could provide cells with the selective advantage that conserves the function of restart proteins despite the fact that suppressor mutations can bypass the need for these proteins. For example, the fact that the helicase motif of PriA is highly conserved among diverse bacteria [43] has been puzzling in light of the fact that its inactivation by the *priA300* mutation seemed to have little effect on the cell phenotype, but the ability of cells with active PriA helicase to grow under conditions that damage DNA at a relatively high rate would indeed be a selective advantage that would conserve this motif.

### The potential defect in restart function promoted by loss of IF2-1 and IF2-2/3

The phenotype of the *<infB(del1)>* mutant raises the question of what IF2-1 could be doing to influence cellular recovery after DNA damage by a PriA helicase-dependent pathway. First, IF2-1 and IF2-2/3 could have different preferences for mRNAs such that IF2-1 specifically promotes the translation of factors needed to support this pathway. Such a mechanism would be novel as such a role of the various IF2 isoforms in promoting differential gene expression has yet to be described. Second, IF2 may act as a transcription factor and the various IF2 isoforms may have different activity in this regard such that IF2-1 is specifically needed to regulate expression of genes needed for PriA helicase-dependent pathways. The finding that IF2 can selectively promote transcription of rRNA by RNA polymerase *in vitro* [26] and the identification of a region in the carboxy terminal region of IF2 with nonspecific DNA binding activity [27] have prompted the proposal that IF2 has activity influencing transcription. Third, IF2 has been shown to have molecular chaperone activity [25]. The IF2 isoforms may ensure that specific factors in their respective pathways are active when required. We have previously proposed a role of IF2 as a chaperone performing a function much like ClpX (Figure 1B–1C) where IF2 binds to a Mu end and prepares the DNA template for assembly of restart proteins, a process beginning with displacement of IF2 from DNA by PriA helicase. The analysis of this present work cannot definitively establish that any one of these possibilities is the basis for IF2's influence on cellular restart mechanisms; however, we favor the third mechanism in which IF2 acts as molecular chaperone, based on the role of IF2 in bacteriophage Mu replication *in vitro* [5], [17], the phenotype of the *infB(del1)* mutant, and the relationship of this allele with *priA300*.

A key question regarding the function of IF2-1 is, why does its loss lead to a severe decrease in viability during growth on MMS despite the fact that the cell remains proficient for supporting replication restart? We suspect that the loss of the preferred IF2 isoform for a restart mechanism, loss of PriA helicase activity, or the complete loss of PriA in the presence of a *dnaC* suppressor results in the inability to fine-tune the progression of restart pathways, a level of regulation that becomes essential when cells must grow under conditions that damage DNA at a high rate. If we speculate that the role of IF2 in Mu replication *in vitro* is applicable for cellular restart mechanisms, we can illustrate the type of regulation that IF2 might exert (Figure 8B).

An important difference between IF2-1 and the truncated forms IF2-2/3 for the assembly of restart proteins at stalled forks may be the mechanism by which they respond to a hypothetical go-ahead signal for restarting DNA replication. When DNA damage is accumulating at a relatively high rate, a mechanism that regulates restart by preventing re-establishment of the replication fork until the template is relatively free of DNA damage may ensure efficient DNA replication in the presence of a DNA-damaging agent. For example, restarting DNA replication before the DNA is relatively free of lesions will only result in the stalling of the fork again, causing delay in establishing a productive replication fork and thus inducing a high level of SOS response that may become toxic.

These considerations are reminiscent of the findings of Flores *et al.* [44], who determined that *priA300* greatly diminishes viability of the *holD^G10^* mutant. The *holD* gene encodes the Psi unit of DNA polymerase III holoenzyme, and the mutant Psi causes frequent replication fork stalling. That is, the effect of *priA300* becomes discernible only when the rate of replication fork stalling becomes high. As noted by Flores *et al.* [44], the deficiency in PriA helicase caused by the *priA300* mutation may lead to the inability to promote duplex opening on the DNA substrate for DnaB helicase loading and replisome assembly [29]; alternatively, another function of PriA besides the helicase could be inactivated by the *priA300* mutation, leading to the inability to cope with frequent fork arrest in the *holD^G10^* mutant. The PriA function needed to sustain high viability of the *holD^G10^* mutant may be related to the pathway in which both IF2-1 and PriA helicase play a role. When cells must grow in the presence of MMS, the action of PriA helicase to displace IF2-1 may play a critical function to ensure maximal cell viability, or conceivably, the inactivation or attenuation of another function by *priA300* may prevent what we call the IF2-1/PriA helicase pathway from operating optimally. This example underscores the possibility that PriA helicase as well as IF2-1 play multiple roles for replication restart, some of which may be part of their mutual participation in the IF2-1/PriA helicase pathway and some of which may not. PriA helicase may play important roles in duplex opening for DnaB loading as well as displacement of IF2 to initiate replication restart, but only the latter may be essential for the IF2-1/PriA helicase pathway.

The role of IF2 isoforms in influencing replication restart mechanisms has important implications for how replication restart and the maintenance of genome stability may be regulated with respect to cell physiology. As a translation factor, IF2 has a strong influence on cell growth and progression through the cell cycle while responding to cellular signals such as the alarmone (p)ppGpp [45], which is an indicator of nutritional deprivation. Depending upon the physiological status, how replication restart is carried out can be critical in determining cell viability, and IF2 may respond to cellular signals to determine the conditions for restart. The IF2 function in translation is a highly conserved one found in all living cells [46], [47]. Its role in influencing pathways for maintaining genome integrity prompts the question whether this general function has been conserved in other organisms to play some function in coordinating replication, recombination, and repair functions with respect to growth conditions.

## Materials and Methods

### Bacterial strains

All experimental analysis was conducted with derivatives of GTN932 (*Hfr del(gpt-lac)5*; see Table S1), an *E. coli* K-12 strain that is a derivative of PK191 [48]. We have conducted PCR and sequencing analysis to verify that this line of strains have wild-type *relA*, not the *relA1* allele [49] as sometimes reported for PK191 strains. The *del(priB)302* and *priC303::kan* alleles from JC19272 [39], *priA2::kan* from PN104 [36], *del(priC)752::kan* from JW0456-1, *del(dnaT)759::kan dnaC(a491t)* from JW4336-2, and *del(argA)743::kan* from JW2786-1 [50] were introduced into bacterial strains by P1*vir* transduction as previously described [39]. Inheritance of *del(priB)752::kan* was screened by PCR analysis with primers PriBupper and PriBlower (Table S2). The *priA300* was introduced by P1 transduction, first transferring the *metB1* allele by selecting for the closely linked *btuB3191::Tn10* from CAG5052; the *priA300* was then transferred from SS97 by selecting for Met^+^ transductants (tetracycline-sensitive transductants were chosen) [19], which were screened by PCR amplification with primers PriA-Nseq and PriA-Cseq and sequenced with revPriA820 primer. The *sulA::*Mu*d(lac,Ap,B::Tn9)* from SS97 [18] or *dinD1::*Mu*d1(lac*,Ap) from PN104 [36] was introduced into strains by P1 transduction and selection on ampicillin plates; transductants were screened for disruption of the *sulA* or *dinD* genes with primers sulAupper and sulAlower or dinDupper and dinDlower, respectively. The *clpX::kan* strain was constructed as previously described [15].

The *del(infB)1::tet* allele was constructed by first integrating a single copy *nusAinfB* operon into a random site of the host chromosome as part of the EZ-Tn5 transposon. The natural *infB* cistron was precisely excised and replaced with a *tet^R^* cistron from pACYC184 [51], using recombineering methods [52] to generate the *del(infB)::tet* allele. As recombination events at the natural *infB* site were very difficult to isolate, we created a PCR template to generate the *del(infB)::tet* allele, with approximately 1-kb of DNA from upstream and downstream of *infB* to flank the *tet* cistron. This template on the pGEM-Teasy vector (Promega) was amplified using *PfuUltra* High Fidelity DNA polymerase (Stratagene) using the primers nusLower and rbfUp2, and the PCR product was used to transform heat-induced DY330 *flgJ::<nusAinfB-kan>*.

The various *flgJ::<nusAinfB-cat>* alleles were constructed by introducing *infB* mutations into the *nusAinfB* operon harbored on the EZ-Tn5 transposon. The transposon was from the pMOD-6<KAN-2/MCS> purchased from Epicentre, and it was introduced into DY330 as a transpososome according to the instructions of the manufacturer. The transposon was determined to be integrated in the *flgJ* gene by a single primer PCR and sequencing method [53]. For introduction of various *infB* alleles at the transposon site, the transposon was modified by recombineering [52]. Heat-induced DY330<KAN-2/MCS> was transformed with a PCR product made by amplifying the *cat* gene of pACYC184 with primers DelMOD6Cat and lowerKanCat (see Table S2). The resulting strain DY330<*del(kan)::cat)*>, which is chloramphenicol-resistant and kanamycin sensitive, serves as the strain for introducing various alleles at this site.

PCR products for introducing the *nusA infB* operon at the transposon were made using pMOD-6<KAN-2/MCS> constructs as template. The *nusA infB* operon, amplified from the *E. coli* chromosome using *PfuUltra* High Fidelity with primers argRmetYp2 and IF2BamHI, was cloned between the SphI and XbaI site of pMOD-6<KAN-2/MCS> (promoter side of the operon is proximal to the SphI site). Various *infB* mutations were introduced into the resulting plasmid. The operon was then amplified using primers lowerMod6Tn and antiSqRP, and the PCR product was used to transform heat-induced DY330<*del(kan)::cat)*>, selecting transformed cells on LB plates containing 25 µg/ml kanamycin and screening for chloramphenicol sensitivity. To construct versions of these *flgJ::<nusAinfB>* alleles that encode chloramphenicol rather than kanamycin resistance, heat-induced DY330 *flgJ::<nusAinfBkan>* strains were transformed with PCR products made by amplifying the *cat* gene of pACYC184 with primers upperKanCat and lowerKanCat. This inactivates the *kan* gene while leaving intact the *nusAinfB* contained within transposons. The resulting constructs were always verified by sequencing as described below.

We could readily knock out the natural *infB* allele of a strain with the <*nusAinfB(wt, del1* or *del2/3)*> cassette by introducing the *del(infB)*::Tet^R^ allele. As the expression of tetracycline resistance was relatively feeble from this allele, introduction of the knockout was most conveniently done by co-transduction with the closely linked *argG*; Arg^+^ transductants of a *del(argG)781::kan* recipient strain co-inherited the *del(infB)*::Tet^R^ allele at a frequency greater than 80%, provided that an *infB* allele which supports cell viability was provided from another site. Even when the second *infB* function was supplied by pSPC*nusAinfB(del2/3,D501N)*, greater than 80% of the Arg^+^ transductants coinherited *del(infB)1::tet* allele, indicating that the multicopy *infB(del2/3,D501N)* can maintain cell viability (the presence of the D501N mutation in the sole *infB* allele was verified by sequencing). When the second *nusAinfB* operon was present on the chromosome, it was introduced into the transposon inserted in *flgJ*. The various *flgJ::<nusAinfB-cat>* alleles were constructed by recombineering methods in DY330 as described above and transferred to other strains by P1*vir* transduction. The *nusAinfB* operon contained within the transposon includes all three ArgR binding sites (see Figure 3A) and extends to the stop codon for *infB*.

As the *nusAinfB* operon in the transposon lacks downstream genes such as *rbf* in the natural operon, the *infB* alleles at the natural site and the transposon in *flgJ* can be separately amplified for DNA sequencing (Figure 3A–3C; primers *p_1_* and *p_2_* for the natural site and *p_1_* and *p_4_* for the transposon site). Thus, the presence of *infB* at the natural site could readily be detected by primers (*p_1_* and *p_2_*) annealing to sites flanking *infB* to yield a 4.7-kb band (Figure 3C, lanes 1, 3, and 9), confirmed by 2.8-kb band yielded by one primer (*p_3_*) annealing within *infB* and one (*p_2_*) downstream of the gene (lanes 2, 4, and 9). (See the list of primers in Table S2.) Knockout of the natural *infB*, in contrast, could be detected with the formation of a 3.2-kb band with primers *p_1_* and *p_2_* (lanes 5 and 7) and no bands (lanes 6 and 8; *cf.* with lanes 2, 4, and 10) with *p_3_* and *p_2_*.

We found this to be the best method for constructing strains with various single-copy *infB* alleles, for the replacement of the wild-type *infB* allele at the natural site proved to be very difficult. As constructed strains were suspected to be potential restart mutants, their *dnaC* allele was sequenced to determine whether any suppressor mutations have accumulated there [39]. None of the mutants we isolated had as severe a phenotype as the *priA* null mutant, and no suppressor mutations in *dnaC* were detected.

### Plasmids

All pSPC*nusAinfB* plasmids with various *infB* alleles were constructed using the pBAD43 plasmid vector (a gift from Dr. Jonathan Beckwith, Harvard Medical School) [54]. This plasmid is a relatively low copy plasmid, having a pSC101 plasmid origin and conferring spectinomycin resistance. The *nusAinfB* operon, amplified by PCR using primers p1nusAinfB and IF2BamHI (see Table S2) and *PfuUltra* High Fidelity DNA polymerase, was inserted into the NsiI-BamHI site of the pBAD43 vector. The *ara* and P_BAD_ sequences required for arabinose-based gene expression by this plasmid were deleted by digestion with NsiI-BamHI and replaced with the *nusAinfB* operon, which begins downstream of the *metYp2* promoter, including the last 5 nucleotides of the Fis binding site and ending with the stop codon for *infB*. As a vector control for the pSPC*nusAinfB* plasmids, pBAD43 was used.

Construction of pBAD24 plasmids [55] that express IF2-1, IF2-2, and S-tagged IF2-2 (S-IF2-2) has been described previously [5]. The plasmid for expressing S-IF2-1 was similarly constructed by amplifying the *infB* gene using primers Stag-IF2-1 and IF2BamHI, which introduce the S-tag coding sequence. The coding sequence was ligated into the NdeI-BamHI site of a pBAD24 vector whose NcoI site has been modified to an NdeI site. The *priB* and *priC* genes were cloned into pBAD24, amplifying these genes using the NdeI-priB/PstI-priB and NdeI-priC/PstI-priC oligonucleotides and ligating into the NdeI/PstI site of the pBAD24 vector.

Site-specific mutagenesis was carried out using the QuikChange Lightning Multi-Site-Directed Mutagenesis Kit purchased from Stratagene, using primers listed for this purpose in Table S2. The *infB(del1)* deletion was generated by amplifying the *nusAinfB* operon harbored on a plasmid vector with 5′-phosphorylated primers delIF2-1UP and delIF2-LOW (see Table S2), with *PfuUltra* High Fidelity and circularizing the linear PCR product with T4 DNA ligase. All mutations were verified by sequencing.

### Immunoprecipitation of IF2-DNA complexes

ChIP analysis was conducted by modification of previously published procedures [56], [57]. The major change was the incubation of cell lysate with 50 µg/ml RNase A at 37°C for 30 min just before the immunoprecipitation step. Additional details are described in Protocol S1.

### Other methods

Sensitivity of strains to MMS was measured both by direct plating on LB plates containing 6 mM MMS and by 15 min exposure to 0–18 mM MMS, the latter based on the procedure by Nowosielska *et al.* [34]. β-galactosidase activity was measured according to the procedure of Miller [58]. Mu was plated on LB plates at 37°C with 10 mM magnesium sulfate on a background of indicator cultures. Mu infective centers from thermoinducible lysogens were plated on a background GTN932 indicator at 42°C. Mu*cts62* lysogens were grown at 30°C. Cultures of *priA2::kan* strains were maintained in Davis minimal medium (Difco) containing glucose, thiamine, proline, and histidine, and the viable count was determined on plates containing the same media.

All results from measuring MMS and UV sensitivity, homologous recombination proficiency, enzyme assays, and Mu plating efficiency are indicated with error expressed as the standard deviation from the mean (at least three independent experiments; the number of independent experiments is indicated). See Protocol S1 for additional details.

## Supporting Information

Figure S1 MMS sensitivity and homologous recombination proficiency of *<infB(del1)>*. A) GTN1050 (*<infB(wt)>*), GTN1114 (*<infB(del1)>*), and GTN1115(*<infB(del2/3>*), which are all derivatives of GTN932 and have the *del(infB)1::tet* allele, were streaked out onto indicated plates. B) Homologous efficiency of the *<infB(del1)*> mutant. GTN1154 (GTN1050 *del(argA)743::kan*), GTN1156 (GTN1114 *del(argA)743::kan*), and GTN1157 (GTN1115 *del(argA)743::kan*) were infected with P1*vir* (AT3327) at a multiplicity of infection of 0.08 PFU/cell (AT3327 is a laboratory strain with an essentially wild-type genotype) and Arg^+^ transductants were scored. The *argA* gene is located at 63.5 min on the *E. coli* map and is not linked to *infB* at 71.4 min as is *argG*. Results (3 independent experiments) are reported relative to the results with GTN1154, which yielded approximately 3000 transductants per ml; the number of transductants were normalized with respect to P1*vir* plating efficiency on each strain as previously described [38]. In all experiments the plating efficiencies on strains being compared were similar, with no more than a 33% variance. C) Complementation of *<infB(del1)>* with pSPC*nusAinfB* plasmids. GTN1156 (*<1>*) and GTN1157 (*<2/3*>) transformed with the indicated plasmids were infected with P1*vir*(AT3327), and Arg^+^ transductants were scored (at least 5 independent experiments). The “*1*” and “*2/3*” refer to the *infB(del1)* and *infB(del2/3)* alleles, respectively, enclosed in “*<>*” to indicate that the allele is present on the transposon.
(TIF)

Figure S2 Sensitivity of restart mutants to 15-min treatment in MMS. A) Activity of PriC expressed in the *<infB(del1)> del(priB)302* genetic background is essential for viability with or without MMS treatment. Strains GTN1514 (pBAD24-priC/*<infB(del1)> del(priB)302*) and GTN1566 (pBAD24-*priC/<infB(del1)> del(priB)302 del(priC)752::kan*) were grown in 0.02% arabinose/LB containing 100 µg/ml ampicillin, treated 15 min with indicated amounts of MMS, and plated on 0.02% arabinose/LB plates. For experiments marked “glucose*”, cultures were grown in plain LB and treated with indicated amounts of MMS, and viability was measured by growth on 0.2% glucose/LB plates. The viable count of untreated cells was also determined by growth on 0.02% arabinose/LB plates. The results are given as the number of colony-forming units scored on glucose plates, expressed as a fraction of the total viable count of untreated cells determined on arabinose plates. Scored on arabinose plates, the viable count of GTN1566 grown in plain LB to OD_600_ of 0.4 was approximately 1×10^8^ cells per ml, at least 50% the viable count of cultures grown to the same OD_600_ in LB containing 0.02% arabinose. The experiments were conducted three times. B) Sensitivity of a *dnaT* knockout mutant to 15-min treatment with MMS. GTN1420, which has the *del(dnaT)759::kan* with the suppressor mutation *dnaC(a491t)*, and GTN932 (WT), which is wild type for these traits, were subjected to treatment with the indicated amounts of MMS, and viability was measured on plain LB plates. The *dnaC(a491t)* allele encodes for DnaC with the D164V alteration, which greatly increases viability of the *dnaT* knockout strain. The experiments were conducted four times.
(TIF)

Figure S3 Mu plating efficiency on various *infB* mutants. Mu*cts62* was titered on the following indicator cultures on LB plates containing 10 mM magnesium sulfate: GTN932, GTN1050, GTN1114, GTN1115, GTN1133, GTN1117, GTN1119, GTN1059, GTN1135, and GTN1137, which have the indicated genotype. Wild-type *priB* (+), *del(priB)302* (−), wild-type *priC* (+), *del(priC)752::kan* (−), *infB(wt, del1, or del2/3)* on the transposon (*<wt>*, *<1>*, and *<2/3*>, respectively). The results are the average of four independent experiments, the error given as the standard deviation from the mean, and are expressed relative to the titer of phage solution on GTN932 indicator, the parental strain that has the wild-type allele for *infB*, *priB*, and *priC*. The typical phage titer on GTN932 was 5×10^9^ PFU per ml. The results are the average of 4 independent determinations with error expressed as the standard deviation from the mean.
(TIF)

Figure S4 Filamentation of *infB* and restart mutants. Unfixed cultures of indicated strains grown in LB to log phase were visualized using a Brightfield Micromaster Infinity Optics Digital Microscope (Fischer Scientific) at 1000X under oil immersion. The white bar indicates a length of 5 µm. A) Combination of *infB* and restart function alleles. Each column of 3 panels is labeled with the *infB* allele in each of the three strains; each row indicates the restart alleles, whether they are *priA300*, *del(priB)302* (PriB^−^), or wild-type. Cultures were grown in LB. The white arrow indicates filaments of moderate length (9–30 µm) for GTN1114, GTN1298, and GTN1323 and filaments >30 µm for GTN1117 and GTN1297. B) The indicated Mu*cts62* lysogens were grown in minimal media to log phase for microscopy. The NIH Image program was used to assist in scoring the number of filaments in various size classes described in the text.
(TIF)

Protocol S1 Additional methods. Further details for the ChIP protocol and the UV and MMS survival analysis are provided.
(DOC)

Table S1 *Escherichia coli* strains.
(PDF)

Table S2 PCR primers.
(PDF)
