# Supplementary figures and images for: A New Role for Translation Initiation Factor 2 in Maintaining Genome Integrity
Source: PLoS Genet. 2012 Apr 19;8(4):e1002648. doi: 10.1371/journal.pgen.1002648 (PMC3334882; doi:10.1371/journal.pgen.1002648)

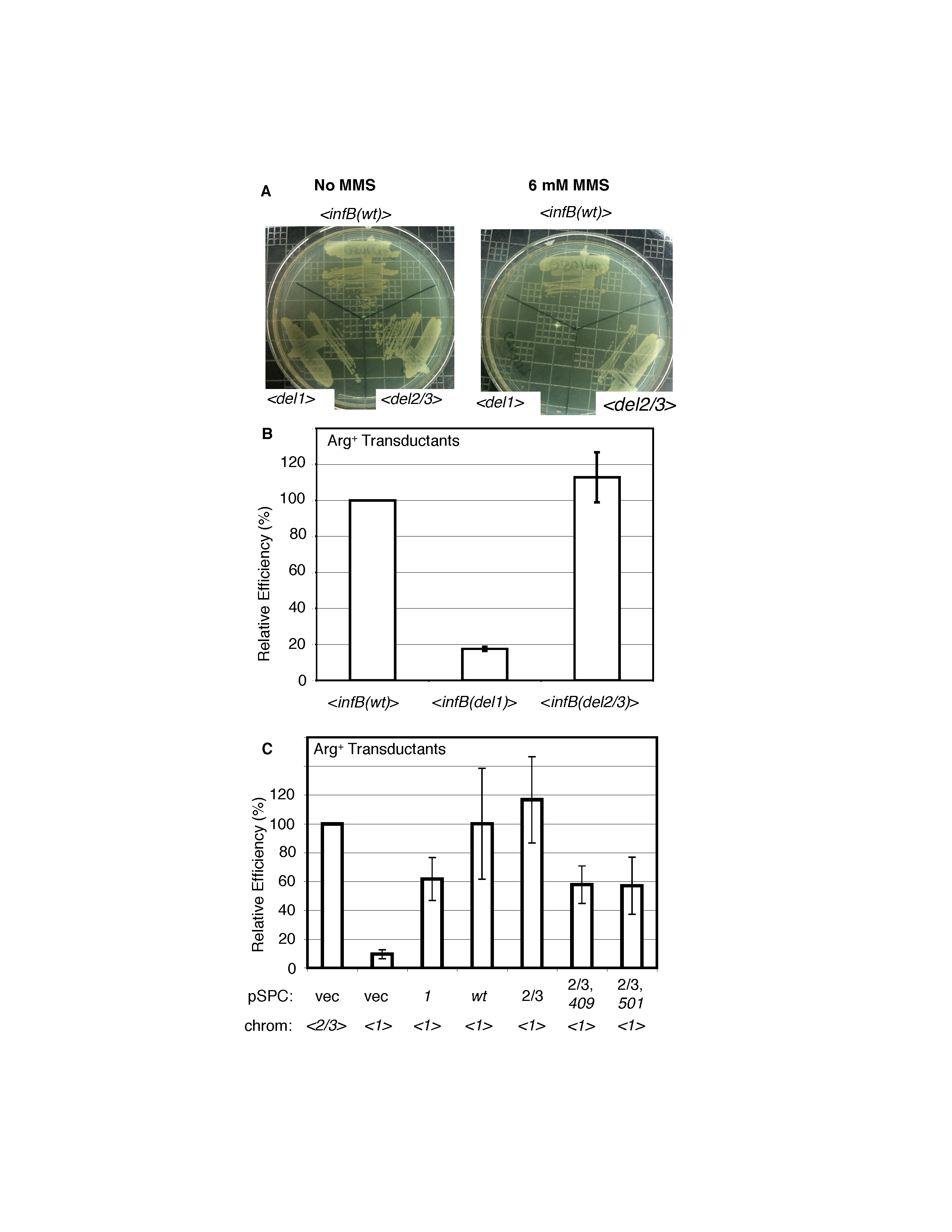

Supplement: Figure S1 — MMS sensitivity and homologous recombination proficiency of <infB(del1)>. A) GTN1050 (<infB(wt)>), GTN1114 (<infB(del1)>), and GTN1115(<infB(del2/3>), which are all derivatives of GTN932 and have the del(infB)1::tet allele, were streaked out onto indicated plates. B) Homologous efficiency of the <infB(del1)> mutant. GTN1154 (GTN1050 del(argA)743::kan), GTN1156 (GTN1114 del(argA)743::kan), and GTN1157 (GTN1115 del(argA)743::kan) were infected with P1vir (AT3327) at a multiplicity of infection of 0.08 PFU/cell (AT3327 is a laboratory strain with an essentially wild-type genotype) and Arg+ transductants were scored. The argA gene is located at 63.5 min on the E. coli map and is not linked to infB at 71.4 min as is argG. Results (3 independent experiments) are reported relative to the results with GTN1154, which yielded approximately 3000 transductants per ml; the number of transductants were normalized with respect to P1vir plating efficiency on each strain as previously described [38]. In all experiments the plating efficiencies on strains being compared were similar, with no more than a 33% variance. C) Complementation of <infB(del1)> with pSPCnusAinfB plasmids. GTN1156 (<1>) and GTN1157 (<2/3>) transformed with the indicated plasmids were infected with P1vir(AT3327), and Arg+ transductants were scored (at least 5 independent experiments). The “1” and “2/3” refer to the infB(del1) and infB(del2/3) alleles, respectively, enclosed in “<>” to indicate that the allele is present on the transposon. (TIF) [file pgen.1002648.s001.tif]

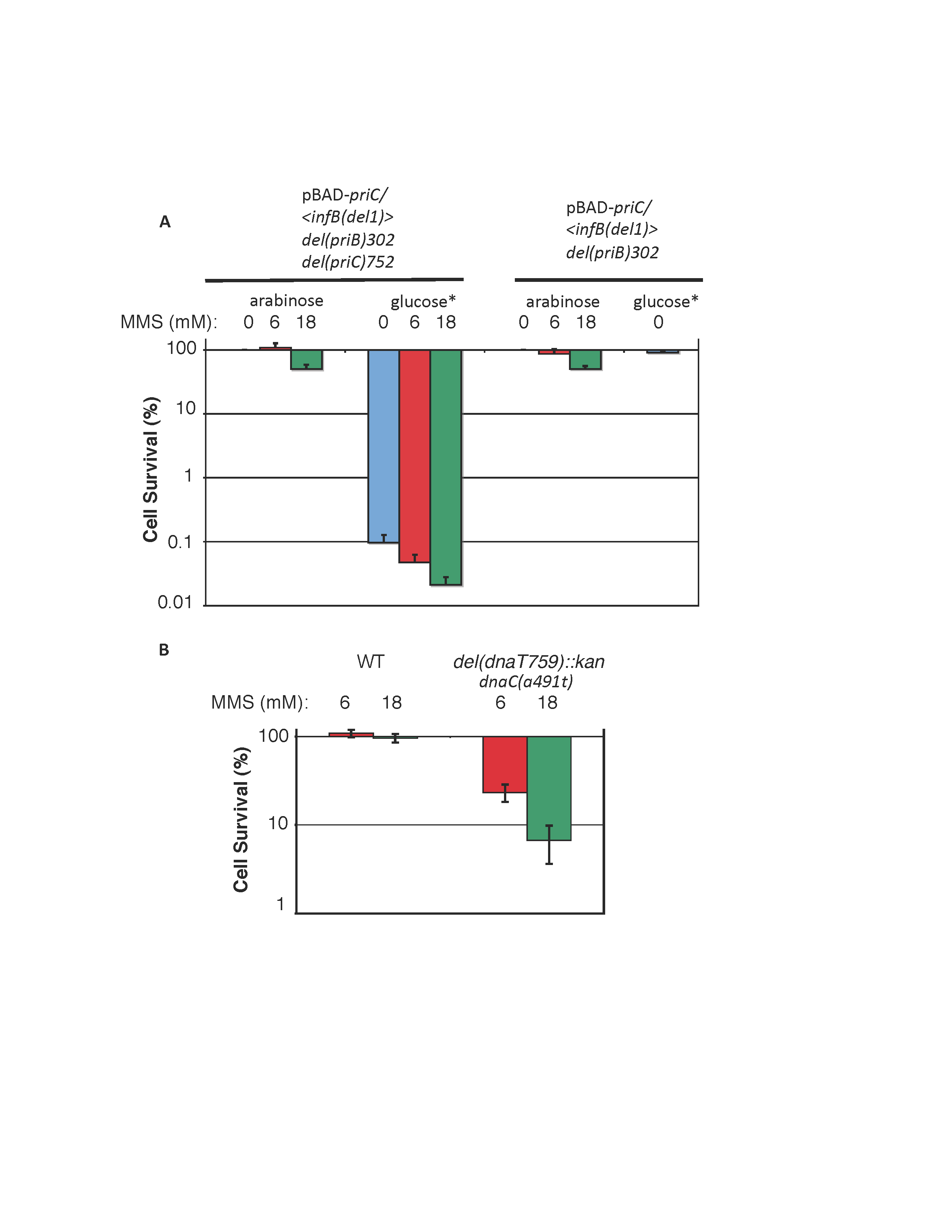

Supplement: Figure S2 — Sensitivity of restart mutants to 15-min treatment in MMS. A) Activity of PriC expressed in the <infB(del1)> del(priB)302 genetic background is essential for viability with or without MMS treatment. Strains GTN1514 (pBAD24-priC/<infB(del1)> del(priB)302) and GTN1566 (pBAD24-priC/<infB(del1)> del(priB)302 del(priC)752::kan) were grown in 0.02% arabinose/LB containing 100 µg/ml ampicillin, treated 15 min with indicated amounts of MMS, and plated on 0.02% arabinose/LB plates. For experiments marked “glucose*”, cultures were grown in plain LB and treated with indicated amounts of MMS, and viability was measured by growth on 0.2% glucose/LB plates. The viable count of untreated cells was also determined by growth on 0.02% arabinose/LB plates. The results are given as the number of colony-forming units scored on glucose plates, expressed as a fraction of the total viable count of untreated cells determined on arabinose plates. Scored on arabinose plates, the viable count of GTN1566 grown in plain LB to OD600 of 0.4 was approximately 1×10^8^ cells per ml, at least 50% the viable count of cultures grown to the same OD600 in LB containing 0.02% arabinose. The experiments were conducted three times. B) Sensitivity of a dnaT knockout mutant to 15-min treatment with MMS. GTN1420, which has the del(dnaT)759::kan with the suppressor mutation dnaC(a491t), and GTN932 (WT), which is wild type for these traits, were subjected to treatment with the indicated amounts of MMS, and viability was measured on plain LB plates. The dnaC(a491t) allele encodes for DnaC with the D164V alteration, which greatly increases viability of the dnaT knockout strain. The experiments were conducted four times. (TIF) [file pgen.1002648.s002.tif]

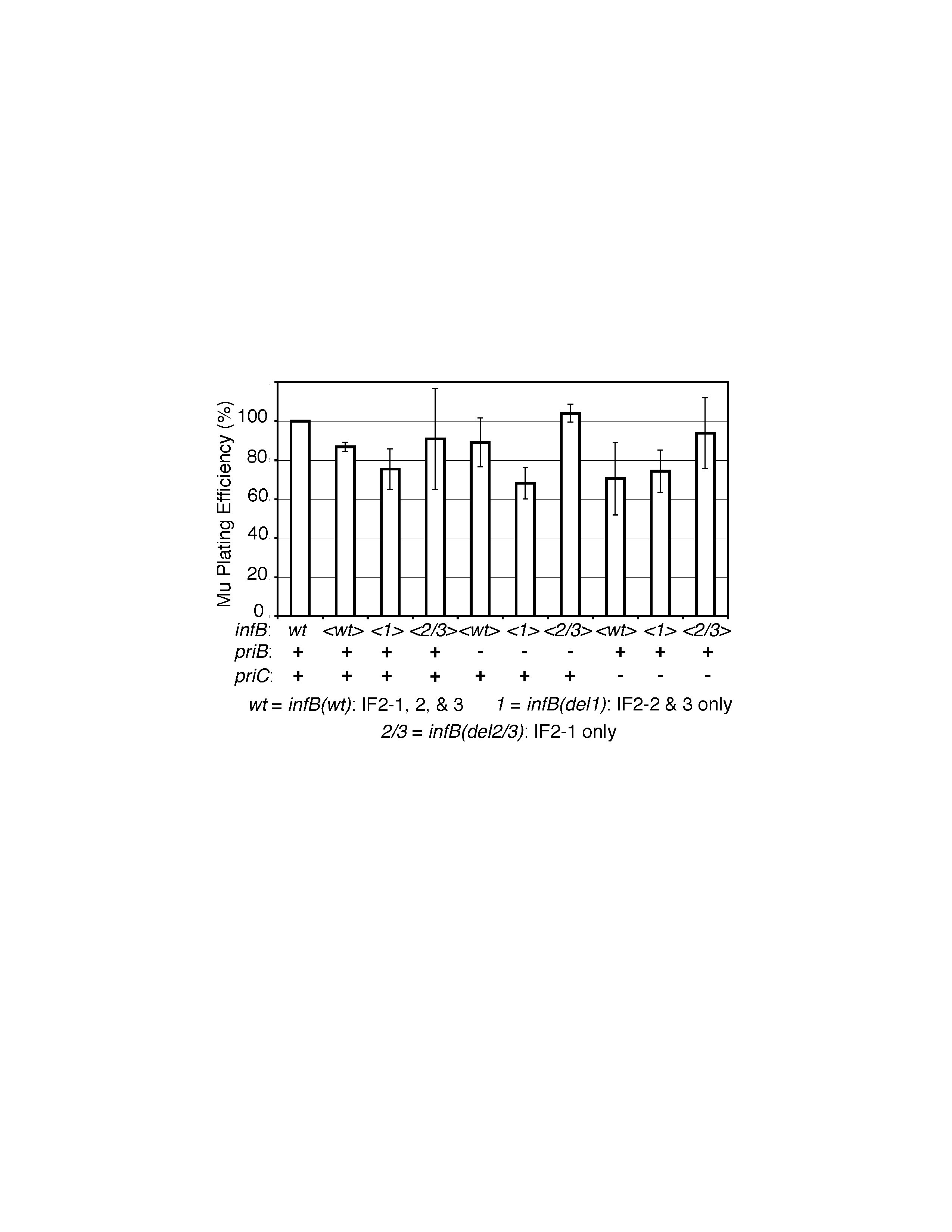

Supplement: Figure S3 — Mu plating efficiency on various infB mutants. Mucts62 was titered on the following indicator cultures on LB plates containing 10 mM magnesium sulfate: GTN932, GTN1050, GTN1114, GTN1115, GTN1133, GTN1117, GTN1119, GTN1059, GTN1135, and GTN1137, which have the indicated genotype. Wild-type priB (+), del(priB)302 (−), wild-type priC (+), del(priC)752::kan (−), infB(wt, del1, or del2/3) on the transposon (, <1>, and <2/3>, respectively). The results are the average of four independent experiments, the error given as the standard deviation from the mean, and are expressed relative to the titer of phage solution on GTN932 indicator, the parental strain that has the wild-type allele for infB, priB, and priC. The typical phage titer on GTN932 was 5×10^9^ PFU per ml. The results are the average of 4 independent determinations with error expressed as the standard deviation from the mean. (TIF) [file pgen.1002648.s003.tif]

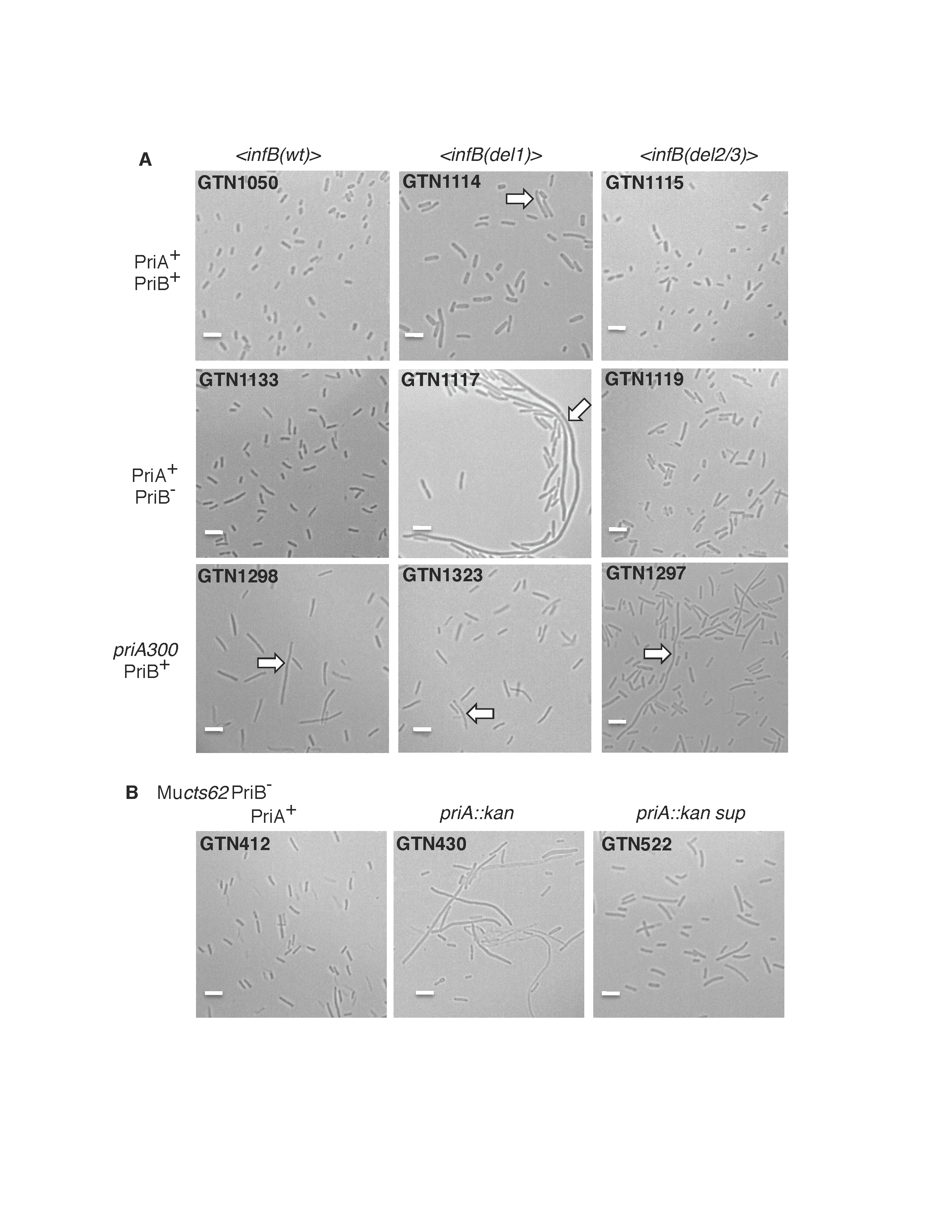

Supplement: Figure S4 — Filamentation of infB and restart mutants. Unfixed cultures of indicated strains grown in LB to log phase were visualized using a Brightfield Micromaster Infinity Optics Digital Microscope (Fischer Scientific) at 1000X under oil immersion. The white bar indicates a length of 5 µm. A) Combination of infB and restart function alleles. Each column of 3 panels is labeled with the infB allele in each of the three strains; each row indicates the restart alleles, whether they are priA300, del(priB)302 (PriB−), or wild-type. Cultures were grown in LB. The white arrow indicates filaments of moderate length (9–30 µm) for GTN1114, GTN1298, and GTN1323 and filaments >30 µm for GTN1117 and GTN1297. B) The indicated Mucts62 lysogens were grown in minimal media to log phase for microscopy. The NIH Image program was used to assist in scoring the number of filaments in various size classes described in the text. (TIF) [file pgen.1002648.s004.tif]
