# Supplementary material for: A New Role for Translation Initiation Factor 2 in Maintaining Genome Integrity
Source: PLoS Genet. 2012 Apr 19;8(4):e1002648. doi: 10.1371/journal.pgen.1002648 (PMC3334882; doi:10.1371/journal.pgen.1002648)
Supplement: Protocol S1 — Additional methods. Further details for the ChIP protocol and the UV and MMS survival analysis are provided. (DOC) [file pgen.1002648.s005.doc]

Protocol S1

for

**A New Role for Translation Initiation Factor 2 in Maintaining Genome Integrity**

K. Elizabeth Madison, Mona R. Abdelmeguid, Erica N. Jones-Foster, and Hiroshi Nakai^*^

Department of Biochemistry and Molecular & Cellular Biology, Georgetown University Medical Center, Rm. 331 Basic Science Bldg., 3900 Reservoir Rd. NW, Washington, DC 20057-1455, USA

**ChIP analysis.** For the capture of IF2-DNA complexes during Mu development, we used lysogens with the *priA300* allele, encoding PriA with the K230R alteration that inactivates PriA’s helicase but keeps intact its role in primosome and replisome assembly by the PriA-PriB pathway . The rationale was to maximize the residence time of DNA-bound IF2, which can be displaced by PriA helicase. Although *priA300* allele was not strictly necessary for the successful capture of the complexes, it nevertheless helped to optimize recovery of Mu DNA-IF2 complexes.

Mu*cts62* lysogens bearing pBAD24 plasmids for expressing S-IF2-1 and S-IF2-2 (and untagged IF2-2 as control) were grown to an OD_600_ of 0.4 in 50 µg/ml ampicillin and 0.02% L-arabinose. 55-ml cultures were then adjusted to a final concentration of 0.2% glucose and 10 mM magnesium sulfate and induced with shaking in a 42°C water bath for 15 min. The temperature was adjusted with ice to 37°C, and incubation was continued. 15-ml of culture were sampled at the onset of induction (0 min) and at various times into the induction. Cells were spun down and resuspended in 1 ml PBS and treated with 1% formaldehyde for 10 min at room temperature. Cells were spun down and washed twice in PBS and resuspended in 500 µl Solution A (10 mM Tris pH 8, 10 mM EDTA, 50 mM NaCl, 20% sucrose) for treatment with lysozyme and addition of 500 µl 2X IP buffer (1X IP is 50 mM Tris pH7, 150 mM NaCl, 5 mM EDTA, 1% Triton X-100) as described . After sonication on ice (10 times for 10 s) and centrifugation to remove cellular debris, RNase A was added to 50 µg/ml, and the mixture was incubated at 37°C for 30 min. 75 µl of the solution was reserved as a sample of total DNA, and the remaining solution was divided in two aliquots, 5 µg of anti-S-tag monoclonal antibody being added to one and none to the other. After a 90-min incubation at room temperature on a rocker, antibody complexes were captured with G-protein Sepharose bead (30 µl settled volume per aliquot), which was previously treated overnight at 4°C in 0.5% bovine serum albumin (Jackson Laboratories, IgG- and protease-free) in 1 X IP buffer. After capture, beads were washed five times in 1 ml 1X IP buffer, three times with 0.5 M NaCl in 1X IP buffer, and twice in TE (10 mM Tris pH 8, 1 mM EDTA). The beads in 50 µl TE and total DNA were adjusted to 0.7% SDS, 0.7 mg/ml proteinase K and incubated overnight at 37°C and 6 h at 65°C. Each DNA sample was recovered in a total of 50 µl 10 mM Tris pH 8.5 using the PCR Purification Kit purchased from Qiagen, adding 500 µl of binding buffer for DNA capture and washing the QiaQuick column with 500 µl of the same buffer before elution. DNA was detected by PCR with Taq polymerase (HotStartIT Taq, Affymetrix), using serially diluted samples as indicated. The primers indicated in Table S2 were used to detect the presence of *E. coli* and Mu sequences in the immunoprecipitate.

**UV and MMS Survival Analysis.** Cultures were grown to OD600 of 0.4 (approximately 2 X 10^8^ cells/ml) in LB. For UV exposure, cells were diluted by adding 2 ml culture to 18 ml 1X Davis salts (Minimal Broth Davis without Dextrose, purchased from Difco) in a 100 X 15 mm petri dish, which was placed on ice for UV exposure and determination of the viable count on LB plates. For examining sensitivity to MMS, cells were either plated on LB plates containing 6 mM MMS or were treated with 6-18 mM MMS for 15 min before determining the viable count on LB plates . For pre-exposure to MMS, 1 ml of culture was spun down and resuspended in 1X Davis containing MMS, which was incubated at 37°C without shaking for 15 min. Each culture was washed once in 1 ml 1X Davis before suspension in same solution for determination of the viable count. For preparation of MMS plates, a 25X MMS solution in LB was first prepared before addition to autoclaved LB/agar solution for pouring plates.

**References**

1. Sandler SJ, McCool JD, Do TT, Johansen RU (2001) PriA mutations that affect PriA-PriC function during replication restart. Mol Microbiol 41: 697-704.

2. Zavitz KH, Marians KJ (1992) ATPase-deficient mutants of the *Escherichia coli* DNA replication protein PriA are capable of catalyzing the assembly of active primosomes. J Biol Chem 267: 6933-6940.

3. Guzman LM, Belin D, Carson MJ, Beckwith J (1995) Tight regulation, modulation, and high-level expression by vectors containing the arabinose P_BAD_ promoter. J Bacteriol 177: 4121-4130.

4. Sokolsky TD, Baker TA (2003) DNA gyrase requirements distinguish the alternate pathways of Mu transposition. Mol Microbiol 47: 397-409.

5. Nowosielska A, Smith SA, Engelward BP, Marinus MG (2006) Homologous recombination prevents methylation-induced toxicity in Escherichia coli. Nucleic Acids Res 34: 2258-2268.
