# Supplementary material for: A New Role for Translation Initiation Factor 2 in Maintaining Genome Integrity
Source: PLoS Genet. 2012 Apr 19;8(4):e1002648. doi: 10.1371/journal.pgen.1002648 (PMC3334882; doi:10.1371/journal.pgen.1002648)
Supplement: Table S1 — Escherichia coli strains. (PDF) [file pgen.1002648.s006.pdf]

Table S1. ***Escherichia coli*** **strains.**

Strain	Relevant genotypes	Source
AT3327	wild type	laboratory strain
CAG5052	*metB1 btuB3191::Tn10*	CGSC[a]
DB1318	*recA938::Tn9-200*	CGSC [1]
DY330	W3110 *del(lacU)169 gal490* λ*cI857 del(cro-bioA)*	D. L. Court [2]
GTN373	GTN932 *priA300 his::*Mu*cts62*	This work
GTN381	GTN932 *priA300*	This work
GTN387	GTN932 *priC303::kan*	This work
GTN394	GTN932 *del(priB)302*	This work
GTN412	GTN932 *del(priB)302 his::*Mu*cts62 dinD1::*Mu*d1(lac*,Ap)	This work
GTN430	GTN412 *priA2::kan*	This work
GTN522	GTN412 *priA2::kan dnaC(g403a)*[b]	This work
GTN622	GTN932 *priA300 clpX::kan his::*Mu*cts62* (GTN373 *clpX::kan*)	This work
GTN932	*del(gpt-lac)5*	This work
GTN1050	GTN932 <*nusAinfB(wt)cat*> *del(infB)1::tet*	This work
GTN1059	GTN932 <*nusAinfB(wt)cat*> *del(infB)1::tet del(priC)752::kan*	This work

GTN1114	GTN932 *<nusAinfB(del1)cat> del(infB)1::tet*	This work
GTN1115	GTN932 *<nusAinfB(del2/3)cat> del(infB)1::tet*	This work
GTN1117	GTN932 *<nusAinfB(del1)cat> del(infB)1::tet del(priB)302::kan*	This work
GTN1119	GTN932 *<nusAinfB(del2/3)cat> del(infB)1::tet del(priB)302::kan*	This work
GTN1133	GTN932 *<nusAinfB(wt)cat> del(infB)1::tet del(priB)302::kan*	This work
GTN1135	GTN932 *<nusAinfB(del1)cat> del(infB)1::tet del(priC)752::kan*	This work
GTN1137	GTN932 *<nusAinfB(del2/3)cat> del(infB)1::tet del(priC)752::kan*	This work
GTN1154	GTN932 *<nusAinfB(wt)cat> del(infB)1::tet del(argA)743::kan*	This work
GTN1156	GTN932 *<nusAinfB(del1)cat> del(infB)1::tet del(argA)743::kan*	This work
GTN1157	GTN932 *<nusAinfB(del2/3)cat> del(infB)1::tet del(argA)743::kan*	This work
GTN1297	GTN932 *priA300 <nusAinfB(del2/3)cat> del(infB)1::tet*	This work
GTN1298	GTN932 *priA300 <nusAinfB(wt)cat> del(infB)1::tet*	This work
GTN1323	GTN932 *priA300 <nusAinfB(del1)cat> del(infB)1::tet*	This work
GTN1376	GTN932 *recA938::Tn9-200*	This work
GTN1384	GTN932 *priA300 sulA::*Mu*d(lac,Ap,B::Tn9)*	This work
GTN1385	GTN932 *sulA::*Mu*d(lac,Ap,B::Tn9)*	This work
GTN1387	GTN932 *<nusAinfB(del1)cat> del(infB)1::tet sulA::*Mu*d(lac,Ap,B::Tn9)*	This work
GTN1396	GTN932 *priA300* Mu*d(lac,Ap,B::Tn9)*$^{+}$ (SulA^{+})	This work

GTN1399	GTN932 <*nusAinfB(del1)cat*> *del(infB)1::tet* Mu*d(lac,Ap,B::Tn9)* + (SulA+)	This work
GTN1420	GTN932 *del(dnaT)759::kan dnaC(a491t)*[b]	This work
GTN1514	GTN1117 pBAD24-*priC*	This work
GTN1566	GTN1117 *del(priC)752::kan* pBAD24-*priC*	This work
GTN1639	GTN1385 *priA2::kan*	This work
PN104	*priA2::kan dinD1::*Mu*d1(lac*,Ap)	CGSC [3]
SS97	*priA300 sulA::*Mu*d(lac,Ap,B::Tn9)*	S. Sandler [4]
JC19272	*del(priB)302 priC303::kan dnaC809*	S. Sandler [5]
JW0456-1	*del(priC)752::kan*	CGSC [6]
JW2786-1	*del(argA)743::kan*	CGSC [6]
JW4336-2	*del(dnaT)759::kan dnaC(a491t)*[b]	CGSC [6]

[a]The Coli Genetic Stock Center, Yale University

[b]The nucleotide replacement in the *dnaC* ORF is indicated.

References

1. Wertman KF, Wyman AR, Botstein D (1986) Host/vector interactions which affect the viability of recombinant phage lambda clones. Gene 49: 253-262.
2. Yu D, Ellis HM, Lee EC, Jenkins NA, Copeland NG, et al. (2000) An efficient recombination system for chromosome engineering in Escherichia coli. Proc Natl Acad Sci U S A 97: 5978-5983.

3. Nurse P, Zavitz KH, Marians KJ (1991) Inactivation of the *Escherichia coli* PriA DNA replication protein induces the SOS response. J Bacteriol 173: 6686-6693.
4. Sandler SJ (2000) Multiple genetic pathways for restarting DNA replication forks in Escherichia coli K-12. Genetics 155: 487-497.
5. Sandler SJ, Marians KJ, Zavitz KH, Coutu J, Parent MA, et al. (1999) *DnaC* mutations suppress defects in DNA replication and recombination functions in *priB* and *priC* double mutants in *E. coli* K-12. Mol Microbiol 34: 91-101.
6. Baba T, Ara T, Hasegawa M, Takai Y, Okumura Y, et al. (2006) Construction of Escherichia coli K-12 in-frame, single-gene knockout mutants: the Keio collection. Mol Syst Biol 2: 1-11.
