# Supplementary material for: A New Role for Translation Initiation Factor 2 in Maintaining Genome Integrity
Source: PLoS Genet. 2012 Apr 19;8(4):e1002648. doi: 10.1371/journal.pgen.1002648 (PMC3334882; doi:10.1371/journal.pgen.1002648)
Supplement: Table S2 — PCR primers. (PDF) [file pgen.1002648.s007.pdf]

Table S2. **PCR primers.**

Primer	Sequence
**ChIP analysis:**	
upperThrAq	ggtacatcagtggcaaatgcagaac
lowerThrAq	cttctaatacgccggccataatgg
upperPyrD	gaagcactggtgcggcagaa
lowerPyrD	accttcggcatctaccagacgaa
upperSerA	acccatctgggcggaagtttg
lowerSerA	caggagaatatcggcctggaagtt
upperMuLq	accgggaggacattggattattcg
lowerMuLq	gttatcggttttgaacgtttttgaagct
upperMuCq	gctggtttaacgccgatgaaatg
lowerMuCq	cagcgcagcaaggcggatt
upperMuRq2	cgattaagcgaggtggacaacga
lowerMuRq2	ctgctttttattcattacatggggatca
**Transposon recombineering:**	
lowerMod6Tn	caaccctgaagctcttgttggctagtgcgta
antiSqRP (p_4)	ttctcgggtgttctcgcatattggctc
DelMOD6Cat	caaccctgaagctcttgttggctagtgcgtagtcgttggcgataccgggaagccctgggccaact
upperKanCat	atgaattgtgtctcaaaatctctgatgttacattgcacaagataccgggaagccctgggccaact
lowerKanCat	aaggacaattacaaacaggaatcgaatgcaaccggcgcaggcaccaggcgtttaagggcaccaat

***infB* cloning:**

p1nusAinfB	tgcatgcatgcacgtctcggtacaccaaatcccagcagtat
argRmetYp2	tcgatgcatgctgagcctggatttccgctctcactgaa
Stag-IF2-1	gcatatgaaagaaaccgctgctgctaaattcgaacgccagcacatggacagcatgacagatgtaa cgattaaaacgctgg
IF2BamHI	gtaaggatccttaagcaatggtacgttggatctcg

***priB* and *priC* cloning:**

NdeI-priB	gggcatgcatatgaccaaccgtctggtgttgtcc
PstI-priB	aaaactgcagctagtctccagaatctatcaattc
NdeI-priC	gggcatgcatatgaaaaccgccctgctgctgg
PstI-priC	aaaactgcagctagcgggttaaacgcgctaacc

Verification of alleles:

nusLower (p_1)	gccatggatatgctgccgcgtgaaaacttc
rbfUp2 (p_2)	aacttcaatgccctgacgcgcata
IF2-2.5'int (p_2)	accgtgacgttgtgatcggcga
DnaC-Nseq	ttccaccaggattcagagggtaacg
DnaC-Cseq	gtgtcgcagtataccgccagaaacgg
PriA-Nseq	ctgatccgcactcttctacggca
PriA-Cseq	gaatttttcgatccgcctcgca
PriBupper	tggtccatcctgaccagagcgaac
PriBlower	gatagcgcgagccagctgacgctg
PriCupper	ccccatttatttctcacttttccgcctcatca
PriClower	ggccgccagcaggataaacggcgtcgtcggtaa

revPriA820	tcaggccggaatgcagaacttc
sulAupper	ccatacagtaactcacaggggctgga
sulAlower	gaaagcattggctgggcgacaa
dinDupper	tcacatggagtgggcaatgaacga
dinDlower	ccttggcttacgcatcgggtgtct

Site-specific mutagenesis:

IF2D409E	gtaaaacctctctgctggaatacattcgttcaacgaaag
IF2D501N	ggttgcagtgaacaagatcaataaaccagaagctgatcc
IF2g474c	gtgaagctgcggaaaaagacaaagtcagcaatcaac
IF2t494c	aagacgatacgactaaaaacgcccaggctg

Construction of *infB(del1)*

delIF2-1UP	aagcgaaacgtgaagctgcggaaaaagac
delIF2-LOW	ctacagtttattacgcttcgtcaccgaa
